# GRP78/BiP determines senescence evasion cell fate after cisplatin-based chemotherapy

**DOI:** 10.1038/s41598-021-01540-8

**Published:** 2021-11-17

**Authors:** Zin Zin Ei, Kanuengnit Choochuay, Alisa Tubsuwan, Decha Pinkaew, Maneewan Suksomtip, Chanida Vinayanuwattikun, Pithi Chanvorachote, Preedakorn Chunhacha

**Affiliations:** 1 Department of Biochemistry and Microbiology, Faculty of Pharmaceutical Sciences, Chulalongkorn University, Bangkok, Thailand; 2 Drugs and Cosmetic Excellence Center, Walailak University, Nakhon Si Thammarat, Thailand; 3 School of Pharmacy, Walailak University, Nakhon Si Thammarat, Thailand; 4 Thalassemia Research Centre, Institute of Molecular Biosciences, Mahidol University, Nakornpathom, Thailand; 5 Division of Cardiology, Department of Medicine, University of Washington, Seattle, WA 98109 USA; 6 Division of Medical Oncology, Department of Medicine, Faculty of Medicine, Chulalongkorn University, Bangkok, Thailand; 7 Department of Pharmacology and Physiology, Faculty of Pharmaceutical Sciences, Chulalongkorn University, Bangkok, Thailand; 8 Preclinical Toxicity and Efficacy Assessment of Medicines and Chemicals Research Clusters, Chulalongkorn University, Bangkok, Thailand

**Keywords:** Cancer, Cell biology

## Abstract

Cisplatin (CDDP) induces senescence characterized by senescence-associated secretory phenotypes (SASP) and the unfolded protein response (UPR). In this study, we investigated the proteins related to the UPR during the senescence cell fate. Strikingly, we found that one of the critical ER-resident proteins, GRP78/BiP, was significantly altered. Here we show that GRP78 levels differentially expressed depending on non-small lung cancer subtypes. GRP78 indeed regulates the evasion of senescence in adenocarcinoma A549 cells, in which the increased GRP78 levels enable them to re-proliferate after CDDP removal. Conversely, GRP78 is downregulated in the senescence H460 cells, making them lacking senescence evasion capability. We observed that the translational regulation critically contributed to the GRP78 protein levels in CDDP-induces senescence. Furthermore, the increased GRP78 level during senescence confers resistance to senolytic drug, Bortezomib, as observed by a twofold increase in IC_50_ in A549 senescence cells compared to the wild-type. This observation is also consistent in the cells that have undergone genetic manipulation by transfection with pcDNA3.1(+)-GRP78/BiP plasmids and pSpCas9(BB)-2A-Puro containing guide RNA sequence targeting GRP78 exon 3 to induce the overexpression and downregulation of GRP78 in H460 cells, respectively. Our findings reveal a unique role of GRP78 on the senescence evasion cell fate and senolytic drug resistance after cisplatin-based chemotherapy.

## Introduction

Lung cancer ranks atop of the new cases and deaths across all cancers combined. In which most of the patients were presented, at the time of diagnosis, at late stages with metastasis either locally or advanced at distant organs^1^. Among various types of lung cancer, non-small cell lung cancer (NSCLC) is considered the majority of lung cancer with approximately 85% of all cases^2^. Since NSCLC is mostly presented at an advanced stage, systemic therapy is considered as a treatment of choice rather than surgical resection. Chemotherapy is beneficial for most of NSCLC patients in combination with radiation or other targeted therapy to improve overall survival^3^.

Among the various types of chemotherapy that is used for NSCLC treatment, cisplatin (cis-diamminedichloroplatinum (II), CDDP) is commonly prescribed^4^. However, a significant proportion of patients have subsequently relapsed in less than 2 years in which they were re-challenged with a platinum-based regimen again would deliver a lower response rate^5^. This was alarming for us to become aware of the negative outcomes whilst treating patients with CDDP.

One of the critical mechanisms underlying cancer relapse from CDDP treatment is related to senescence, also refers to therapy-induced senescence (TIS). CDDP has been found to induce cellular senescence in several cancers including ovarian^6^, melanoma^7^ as well as head and neck cancer^8^. Indeed, these senescence cells may also produce their progeny with more aggressive phenotypes^9,10^. Then, the extensive research to investigate the senescence mechanisms is required for reducing the chance of relapse and ultimately reduces cancer-related deaths.

CDDP triggers senescence via DNA damage response, in which the activation of p53 and other CDKi is implemented. In melanoma cells, CDDP treatment induces robust senescence-associated genes including p53, p16 as well as DNA damage markers such as γH2AX, DDB2^7^. Also, these senescence cells elicit non-cell-autonomous effects by producing a mixture of either soluble and insoluble factors which can be referred to as senescence-associated secretory phenotype (SASP). This SASP is the term that is used to define the senescence cells that secretes a combination of cytokines, chemokines, growth factors, extracellular matrix proteases, and other signaling molecules. Since SASP is an adaptive process that involves the dramatic increase in protein synthesis, indeed this phenomenon evokes the unfolded protein response (UPR) in the endoplasmic reticulum (ER)^11^. After monitoring the UPR associated proteins during senescence cell fate, we found that the critical ER residence protein GRP78 is most robustly altered during this process. In line with previous reports, many of them reported that GRP78 was mostly upregulated in various senescence models, although some of them were downregulated^12^. In addition, based on the information from the SeneQuest database, https://senequest.net/, a well-known source of senescence-associated gene information, it was found that GRP78 is increased in 2 studies whereas downregulated in 4 studies. Indeed, when considering in the context of CDDP induces senescence, GRP78 was downregulated in the ovarian cancer model, A2780^6^. However, this finding was performed in a single cell line in which the extrapolation into more diverse types of cancers may be limited. In this study, we demonstrated that during the senescence cell fate, GRP78 may be expressed differentially in a different subtype of NSCLC cell lines. This differential expression contributed to a different ability to evade senescence as well as the response to the senolytic drug, Bortezomib. This finding provides more insight into the role of GRP78 in senescence cells, in which it might be a potential target to eliminate senescence cancer cells.

## Results

### Cisplatin (CDDP) induces senescence-like phenotype in non-small cell lung cancer cell lines

**Figure 1:**
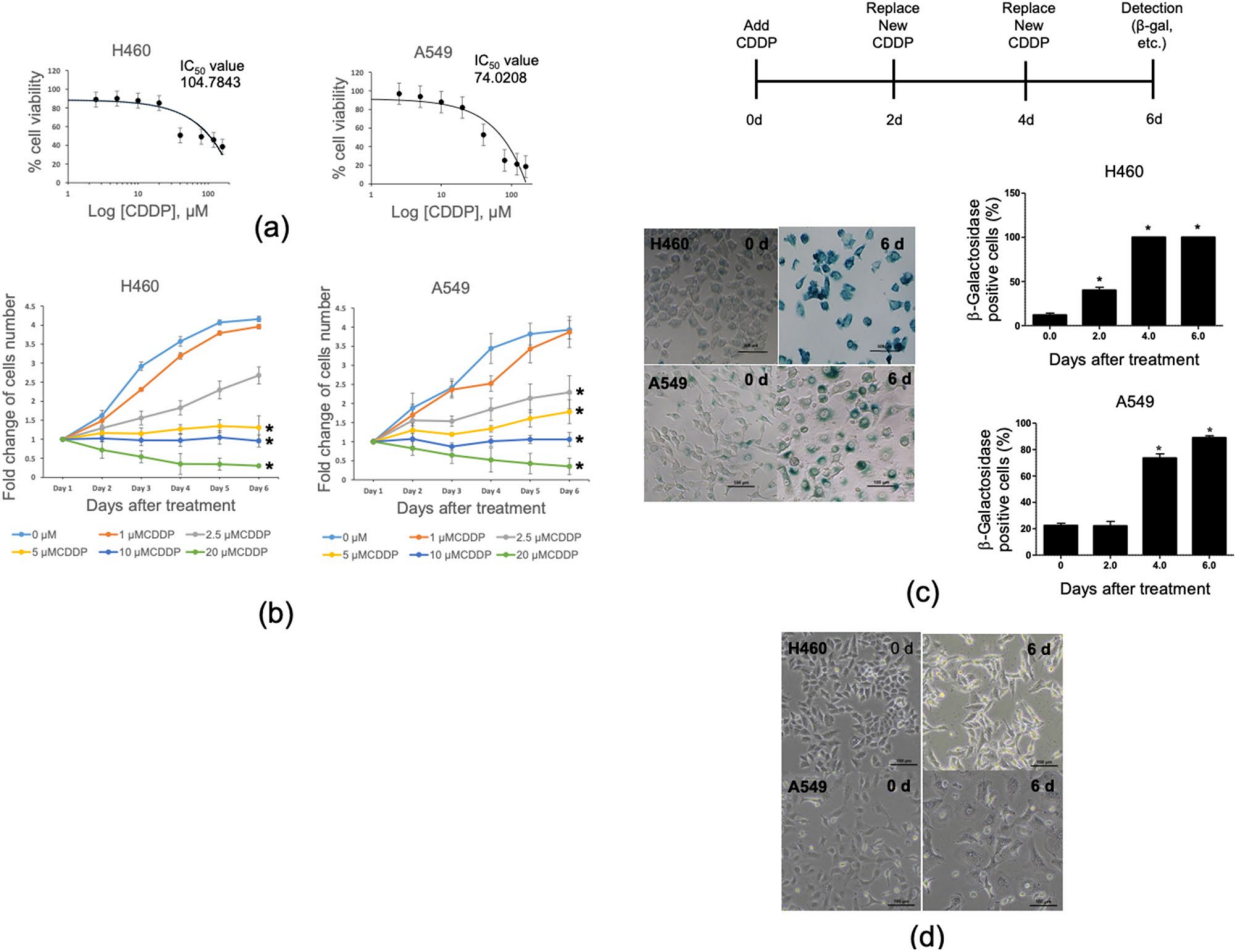
CDDP induced senescence cell fate in H460 and A549 (**a**) IC_50_ values of H460, A549 cell line obtained from exposure to CDDP (2.5–160 µM) for 24 h treatment. (**b**) Cell proliferation curves of H460, A549 cell lines were exposed to CDDP (1–20 µM) for 6 days. (initial cell concentration is 1.5 × 10^3^ cells/well) (**c**) Time-course analysis of senescence-associated β-galactosidase in H460, A549 cells treated with CDDP at 5 µM, the percentage of β-gal blue staining positive cells were calculated (n = 3). (**d**) NSCLC morphology analyzed following 6 days of treatment with CDDP at 5 µM. Data in (**b**) are representative curves of at least three independent experiments. For other data, the average of three independent experiments is shown. Data was analyzed by two-tailed Student’s t-test. *Denotes *p* < 0.05. The scale bar in (**c**) and (**d**) is 100 µm.

Cellular senescence has been shown to cause cancer resistance to drugs and the conviction by which such senescence is being researched. Cisplatin (CDDP) is considered as the first-line agent for lung cancer treatment, however, the emergence in the resistance due to TIS is inevitably occurred and most likely involved with the recurrence, refractory tumor that ultimately becomes difficult to treat. To assess the phenotypic characteristic of CDDP resistance lung cancer cells, we enriched the resistance population by prolonged CDDP treatment since it promotes DNA repair capacity and possess an increased genomic alteration frequency^13^. We firstly investigate the IC_50_ of CDDP in 2 NSCLC cell lines including H460, A549 cells by treating the cells with various doses of CDDP and measuring the cell viability after 24 h treatment (Fig. 1a). The resulted IC_50_ for H460, A549, were 104.78, 74.02 μM. respectively. These IC_50_ are considered within the range reported in the Genomics of Drug Sensitivity in Cancer Database for lung cancer (2). Since our strategy is to use a prolonged sub-toxic dose of CDDP treatment in NSCLC cell lines, we next examined the cell proliferation curve in the sub-toxic dose range over a 6 day period (CDDP6d) (Fig. 1b). Among various doses of CDDP tested, CDDP 5 μM causes the cell proliferation inhibition over the 6 days in two cell lines used in this study, suggesting that they are all of comparable sensitivity to CDDP (Fig. 1b).

To ascertain that the CDDP6d populations were senescence-like phenotypes, we stained CDDP6d with a β-galactosidase (β-gal) staining kit. As shown in Fig. 1c, the percentage of β-galactosidase positive cells were increased dramatically starting from day 2 and reaches maximum at day 6 post CDDP treatment almost reaching 100% β-galactosidase positive cells. In addition to β-gal staining, we observed morphological enlargement in CDDP6d in all cell lines tested, suggesting a senescence-like phenotype (Fig. 1d). Taken together, CDDP treatment at 5 μM inhibits cell proliferation in H460, A549 upon 6 days of treatment with CDDP which was characterized by positive β-gal staining and cell enlargement, suggesting a senescence cell fate response.

### CDDP induces H460 senescence through DNA damage response via p53/p21 and pathway and induces cell cycle arrest at G_2_/M phase

**Figure 2:**
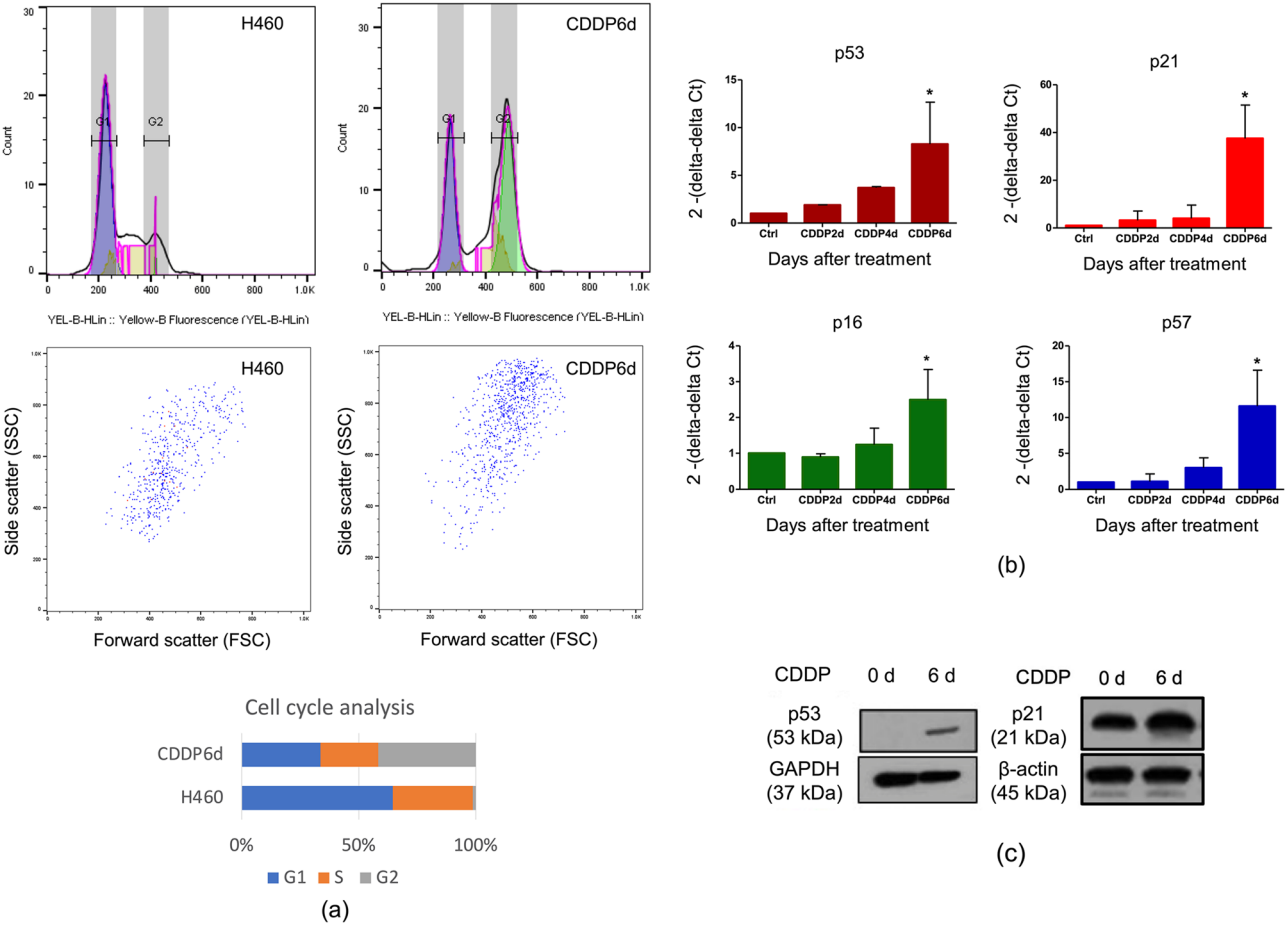
CDDP-induced H460 senescence cell through DNA damage response via p53/p21 pathway. (**a**) Cell cycle analysis after 6 days of treatment of 5 µM CDDP in H460 cells by flow cytometry (**b**) Time-course analysis of the relative mRNA levels of p53, p21, p16 and p57 by real-time Q-PCR in H460 cells treated with CDDP at 5 µM. (**c**) p53, p21 Western blot analysis of CDDP induced H460 senescence cells (the full-length blots are presented in Supplementary Figures). For all the data, the average of three independent experiments is shown. Data was analyzed by two-tailed Student’s t-test. *Denotes *p* < 0.05.

To further confirm the cell cycle arrest during senescence, we selected H460 for detailed characterization on the cell cycle by using flow cytometry and the real-time RT-PCR for the senescence markers. For cell cycle analysis, we detected the accumulation of G_2_/M phase subpopulation at 48.4% of the total cell population compared to 1.36% in the CDDP after 6 days of treatment and control cells, respectively (Fig. 2a). This suggested that CDDP induces DNA damage in which the cell cycle was arrested at G_2_/M checkpoint.

Cyclin-dependent kinase inhibitors (CDKi) are critical regulators during the DNA damage repair process^14^. To investigate the mechanisms underlying cell cycle checkpoint induced by CDDP in H460 cells, we employed the qRT-PCR analysis to identify the level of p53 transcripts together with the key CDKi and identified that p53 mRNA, p21 mRNA (CDKN1A) were significantly upregulated from day 2–6 in H460 cell line, whereas, p16 mRNA (CDKN2A) was slightly increased during the senescence cell fate (Fig. 2b). In addition, we found p53 protein level was increased in CDDP6d (Fig. 2c). Since the p53/p21 pathway relies heavily on TP53 mutation status, the increased level of p53 and its transcriptional target p21 are expected in H460 which carries wild type p53^15^. We also detected the robust expression of p57 mRNA (CDKN1C) which is a potent tight-binding of several G_1_ cyclin/CDK complexes (cyclin E-CDK2, cyclin D2-CDK4, and cyclin A-CDK2) during the senescence. 

Interestingly, all these CDKi mRNAs were upregulated to the highest extent on day 6 similar to the extent of β-galactosidase positive cells. Taken together, the CDDP treatment initially triggers H460 senescence on day 6 via p53/p21 related mechanism.

### CDDP-induced senescence-associated secretory phenotype (SASP) and non-cytoprotective unfold protein response in H460

**Figure 3:**
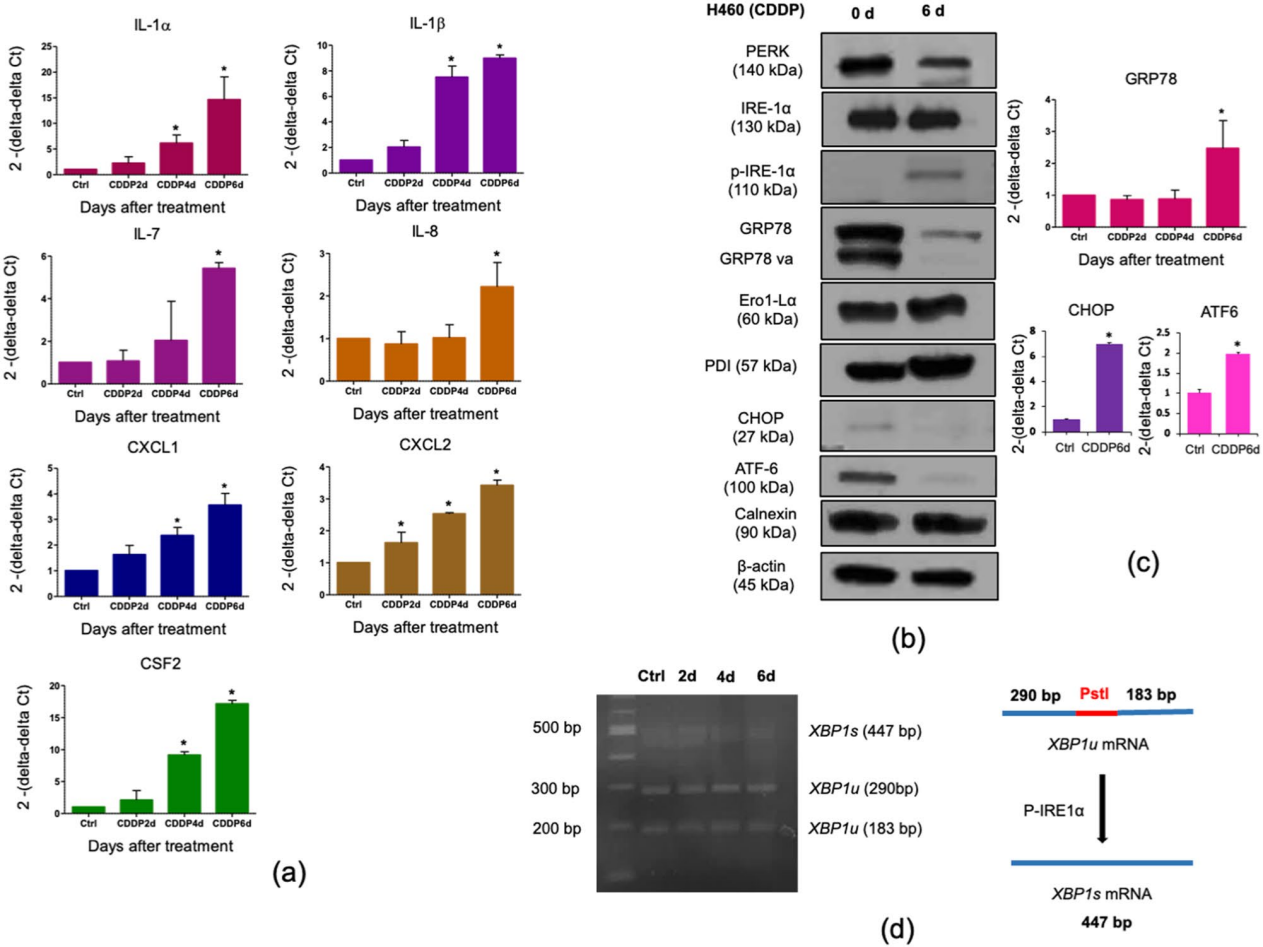
CDDP-induced SASP in H460 and non-cytoprotective unfolded protein response. (**a**) Time-course analysis of the relative mRNA levels of various SASP genes by real-time Q-PCR in H460 cells treated with CDDP at 5 µM. (**b**) Protein expression levels of ER stress markers were detected in CDDP induced H460 senescence cells during SASP by western blot analysis (the full-length blots are presented in Supplementary Figures) (**c**) Time-course analysis of the relative mRNA levels of GRP78, CHOP and ATF-6 by real-time Q-PCR in H460 cells treated with CDDP at 5 µM. (**d**) H460 cells were treated with 5 µM CDDP at the respective days indicated. The cDNA from total RNA from these cells was subjected to PCR and *Pst1* restriction digestion (CTGCA|G) to semi-quantitatively evaluate the levels of *XBP1s*. *XBP1u* unspliced *XBP1*, *XBP1s* spliced *XBP1*. For all the data, the average of three independent experiments is shown. Data was analyzed by two-tailed Student’s t-test. *Denotes *p* < 0.05.

Remarkably, senescent cells secrete various cytokines, chemokines, and proteinases that are critically implicated in cancer progression^16–18^. We further explore whether the senescence H460 cells developed SASP by monitoring the mRNA levels of several SASP genes. As expected, we observed that most of the SASP mRNA including IL-1α, IL-1β, CSF2, robustly expressed during the CDDP treatment and reached the maximum on day 6, whereas some of the SASP mRNAs such as IL-7, IL-8, CXCL1, and CXCL2 were slightly increased (Fig. 3a). Furthermore, we cultured wild type H460 cells (wtH460) with the conditioned medium taken from H460 with CDDP treatment at days 2, 4, and 6, which were designated as H460-CDDP2d, H460-CDDP4d and H460-CDDP6d, respectively. Indeed, we found that only the conditioned medium taken from H460-CDDP6d induces wtH460 proliferation significantly. However, this finding was not observed on the conditioned medium from H460-CDDP2d, H460-CDDP4d, A549-CDDP2d, A549-CDDP4d, and A549-CDDP6d upon treatment to the wild type H460 and A549 cells (Supplementary Fig. S1G–H).

Indeed, the adaptive response to the increased protein synthesis required during SASP could potentially induce the unfolded protein response (UPR) in the endoplasmic reticulum (ER). We then characterized the UPR associated proteins to test whether the UPR is activated during SASP. As shown in Fig. 3b, we observed that ERO1-Lα, PDI, IRE-1α, and calnexin were found unchanged during SASP. Strikingly, the ER-resident chaperone, GRP78 protein was found strongly downregulated whereas GRP78 mRNA is slightly increased during SASP (Fig. 3c), suggesting a translational regulation of GRP78 during senescence fate. In addition, p-IRE-1α was increased during SASP suggesting a mild to moderate ER stress. In addition, ATF-6 which is a transmembrane glycoprotein of the ER, was being undetectable with our antibody that recognizes the total ATF-6 protein (Fig. 3b). In fact, ATF-6 is cleaved liberating an amino-terminal fragment that further translocates to the nucleus during ER stress^19^. Since GRP78 reduction, IRE-1α activation and ATF-6 cleavage usually resulted in ER stress and may subsequently induced apoptosis^20,21^. We then performed the western blot analysis to investigate whether CHOP protein was altered during this phenomenon. Indeed, we found that the pro-apoptotic protein CHOP is absence (Fig. 3b), whereas the CHOP transcription was increased during SASP (Fig. 3c). We then speculated that all these ER stress regulating genes including GRP78, CHOP has a translational control during stress condition as previously suggested^22^ . Due to CHOP protein expression is not pronounced, this modest ER stress in SASP is less likely to promote ER stress induced apoptosis. To further characterize the UPR in SASP, we employed the *XBP-1* splicing assay to confirm whether the upregulation of p-IRE-1α may be involved with *XBP-1u* mRNA splicing during SASP. Since upon IRE-1α phosphorylation, p-IRE-1α ultimately oligomerized, enabled the endoribonuclease activities in which *XBP-1* mRNA further gets cleavaged by p-IRE-1α, resulting in *XBP-1 s* mRNA. Indeed, we observed the rate of *XBP-1* splicing remains unaffected by SASP in contrast to the increasing of p-IRE-1α (Fig. 3b,d). This finding suggested that CDDP induced SASP with UPR activation through p-IRE-1α upregulation. However, this UPR activation is independent of *XBP-1* splicing in concomitant with the critical ER-resident chaperone protein, GRP78, is strongly abolished.

### GRP78 expression during senescence promotes senescence evasion upon CDDP removal

**Figure 4:**
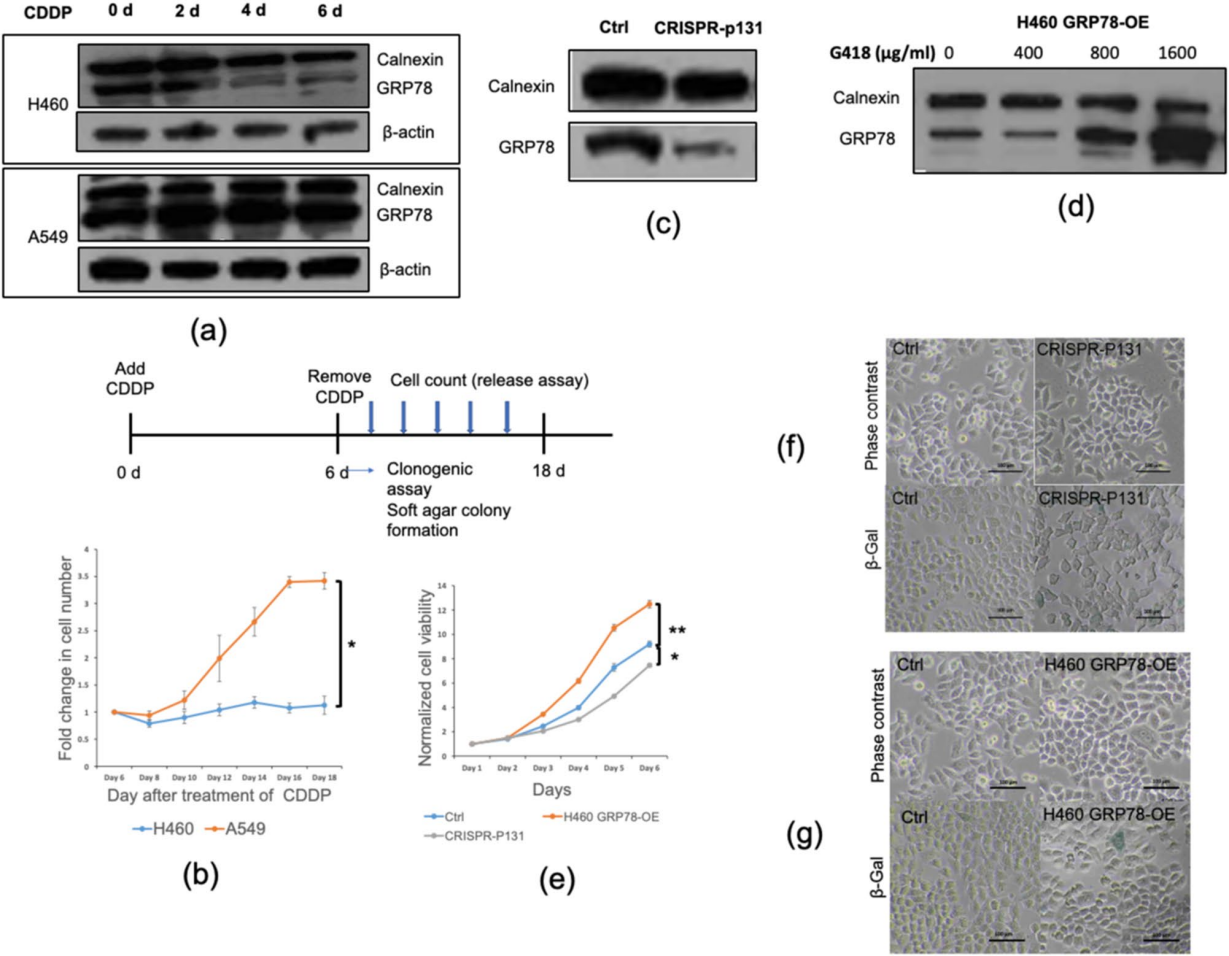
GRP78 primes the senescence cells for cell cycle re-entry when CDDP was removed. (**a**) Time-course analysis of the GRP78 protein expression in H460, A549 cells treated with CDDP at 5 µM. (**b**) Representative proliferation curves of H460, A549 cells treated with CDDP for 6 days then untreated for the remaining days (release) until day 18 (**c**) GRP78 Protein levels in H460 CRISPR-P131 cells by western blot analysis. (**d**) GRP78 Protein levels in H460 GRP78-OE cells selected by G418 (the full-length blots are presented in Supplementary Figures). (**e**) Proliferation assay of H460 CRISPR-P131, H460 GRP78-OE cells compare with control by monitoring for consecutive 6 days. (initial cell concentration is 10^3^ cells/well) (**f,g**) Cell morphology and senescence-associated β-galactosidase staining of H460 CRISPR-P131 and H460 GRP78-OE cells. Data was presented as representative curves of at least three independent experiments. The scale bar in figure (**f,g**) is 100 µm. *Denotes *p* < 0.05, **Denotes *p* < 0.01.

Since the GRP78 protein downregulation is observed in H460-CDDP6d, we next confirm this finding in another NSCLC model, A549. Interestingly, we found that GRP78 protein levels were stable during the course of CDDP treatment in A549 in contrast to H460 (Fig. 4a). Indeed, the A549 GRP78 mRNA transcription is tenfold increased during senescence (Supplementary Fig. S1) compared to 2.5-fold increased in H460 GRP78 transcripts (Fig. 3c). To further test the role of GRP78 during senescence on the specific characteristic including reversible senescence, cancer stem cells-like phenotype as well as EMT, we then performed the release assay, clonogenic assay, and anchorage-independent growth to measure those abilities accordingly.

Senescence was previously recognized as a terminal arrest of cell division, however, compelling evidence suggested that the senescence can be reversible, especially the senescence-associated from chemotherapy^23–25^. To test whether the presence of GRP78 during senescence may involve the reversible senescence, we performed the release assay in A549 versus H460. Upon CDDP removal on day 6, we observed a significant increase in A549 cell proliferation but not for H460 (Fig. 4b) until 18 days after the CDDP treatment. Suggesting that GRP78 may contribute to the reversible senescence phenotype.

As GRP78 may contribute to the reversible senescence, we further clarify the role of GRP78 in this matter by using CRISPR/Cas9 gene manipulation targeting exon 2, 3 of the GRP78 gene. After clonal selection, we successfully generated GRP78 downregulated cells, named as CRISPR-P131, in which the protein level was subsequently analyzed (Fig. 4c). Also, we have generated GRP78 overexpressing cells by pcDNA3.1(+)-GRP78/BiP plasmids transfection, name as H460 GRP78-OE (Fig. 4d). As expected, CRISPR-P131 cells have extremely slow proliferation whereas H460 GRP78-OE cells proliferate faster (Fig. 4e). We also performed β-gal to check whether these cells had undergone senescence, however, there was no significant increase in β-gal positive cells in CRISPR-P131 and H460 GRP78-OE cells suggesting that the lost or gain of GRP78 levels only affected cell cycle progression but it does not promote senescence per se (Fig. 4f,g). Taken together, GRP78 promotes cell cycle re-entry in the senescence cells but the lack of GRP78 expression only perturb cell proliferation without affecting senescence.

Since the senescence is associated with cellular reprogramming, in which the integrated signals manipulate the phenotype of the cells resulting in a more virulent characteristic compared to their predecessors. The reprogramming during senescence was described either as epithelial to mesenchymal transition (EMT) or cancer stemness, we then test whether H460-CDDP6d is associated with both phenomena. Firstly, we investigate the mRNA transcript of EMT markers including E-cadherin, N-cadherin, Slug, and Vimentin. We found that E-cadherin mRNA is increased during SASP cell fate, whereas N-cadherin, Slug, and Vimentin mRNA were slightly altered (Supplementary Fig. S2). Taken together, our results suggested that H460-SASP is not associated with EMT. Furthermore, we performed the western blot analysis of the cancer stem cell marker, ALDH1A1. We observed that ALDH1A1 in H460-SASP was reduced, suggesting that H460-SASP is not associated with cancer stem cells (Supplementary Fig. S3). In addition, we performed soft agar colony formation and clonogenic assay in H460-CDDP6d and A549-CDDP6d to further confirm the anchorage-independent growth and stemness phenotype. As expected, these senescence cells lacked EMT and stemness characteristics since the number and size of colony formed were less detected than control cells (Supplementary Figs. S4–S5). We also investigated the GRP78 expression in other cell lines including H23, H292 and patient-derived Primary Lung Cancer Cell Line (ELC16, ELC20). We observed a differential GRP78 expression during senescence among these cells in which H23, H292 have sustained GRP78 levels whereas ELC16, ELC20 exhibit GRP78 downregulation (Supplementary Fig. S6).

Taken together, though senescence from H460-CDDP6d and A549-CDDP6d have a different GRP78 level during senescence, however, this difference may not seem to have an impact on the clonogenic assay and soft agar colony assay, suggesting that GRP78 may not be involved in these phenomena but GRP78 during senescence prime the senescence cells for cell cycle re-entry when the drug had been removed.

### GRP78 expression during senescence protects the cells from Bortezomib treatment

**Figure 5:**
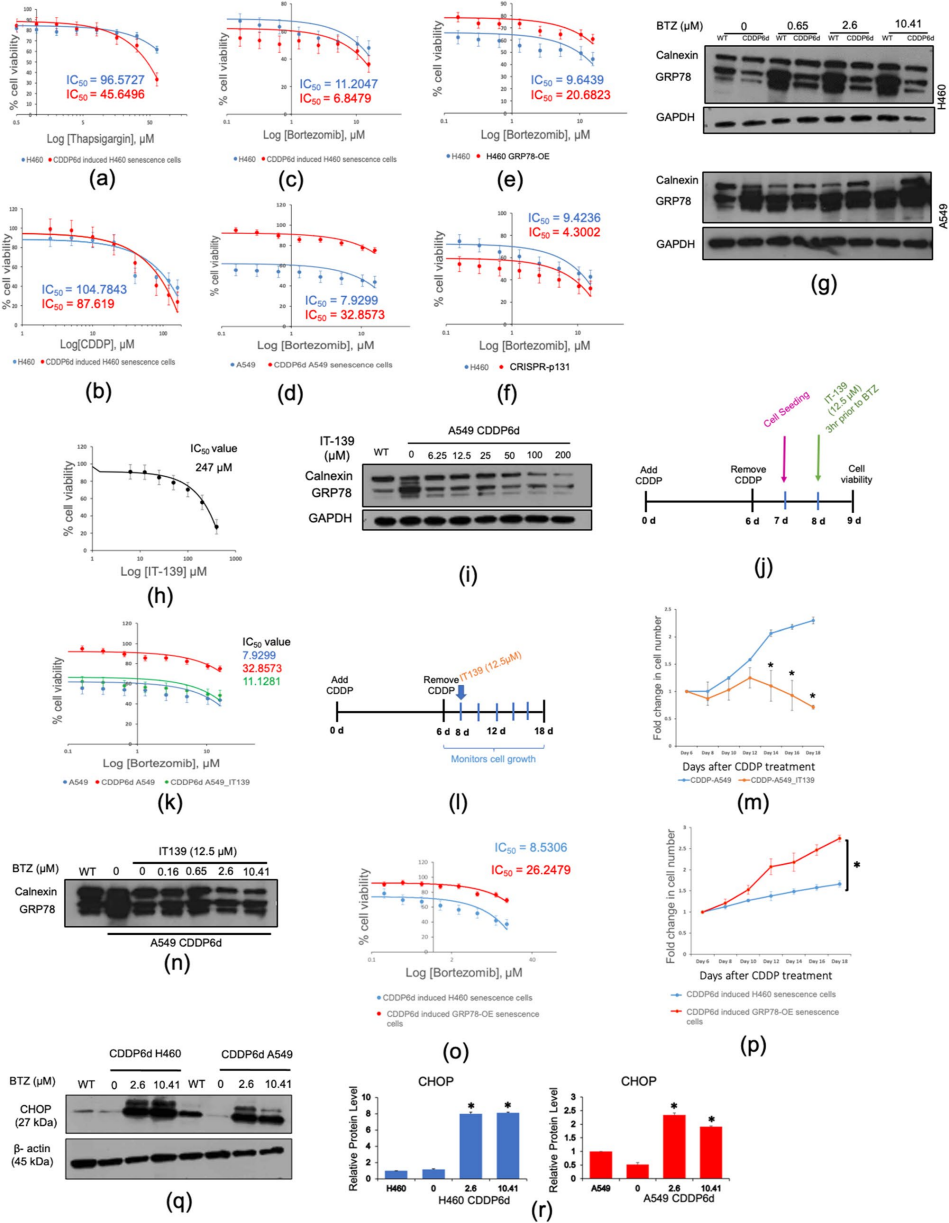
GRP78 is required for Bortezomib (BTZ) resistance senescence cells. (**a–c**) IC_50_ value for H460 cells and CDDP6d cells with different concentration of (**a**) Tg (0.5–64 µM). (**b**) CDDP (2.5–160 µM). (**c**) Bortezomib (0.16–15.62 µM). (**d**) Bortezomib in A549. (**e**) Bortezomib in H460 GRP78-OE (**f**) Bortezomib in CRISPR-P131 (**g**) The level of GRP78 protein expression in A549 and H460 cells treated with BTZ at indicated concentrations. (**h**) IC_50_ value for A549 treated with IT-139 (6.25–200 µM). (**i**) The level of GRP78 protein expression in A549-CDDP6d in the presence of IT-139 at indicated concentrations. (**j**) IT-139 treatment scheme for cell viability. (**k**) IC_50_ value of Bortezomib treated in WT A549, A549-CDDP6d and A549-CDDP6d pretreated with 12.5 μM IT-139. (**l**) IT-139 treatment scheme for cell proliferation. (**m**) Cell proliferation of A549-CDDP6d with IT-139 treatment versus control. (**n**) GRP78 protein expression in A549-CDDP6d after Bortezomib treatment in the presence of IT-139 (the full-length blots are presented in Supplementary Figures). (**o**) IC_50_ value of Bortezomib treated in H460-CDDP6d versus H460-GRP78-OE-CDDP6d. (**p**) Cell proliferation of H460 CDDP6d versus H460 GRP78-OE-CDDP6d after CDDP removal. (**q**) The level of CHOP protein expression in H460-CDDP6d and A549-CDDP6d in the presence of Bortezomib at indicated concentrations. (**r**) Comparison of CHOP protein expression from the band intensity relative to their respective control normalized with β-actin. For all the data, the average of three independent experiments is shown. Denotes *p* < 0.05.

Since the tumor recurrence was found to be associated with the cancer senescence cell burden^26–29^. The effective therapy is urgently needed to reduce the cancer senescence cell population in patients aiming to increase progression-free survival. Several promising agents have been reported to reduce senescence burden, also known as senolytic drugs, providing alternative strategies to reduce cancer progression^30,31^. As senescence cells secrete an array of inflammatory cytokines and chemokines as shown in Fig. 3a and Supplementary Fig. S1, together with the downregulation of GRP78 and increasing in p-IRE-1α (Fig. 3b), we speculated that the ER of these senescence cells should be easily perturbed. Since the ER homeostasis is tightly controlled, the ER stress level that goes beyond a certain point can shift from pro-survival to the proapoptotic pathway, culminating in ER stress induced apoptosis. As H460-SASP has a modest ER stress level, we hypothesize that further increase in the ER stress should promote ER stress and induce apoptosis. To test this hypothesis, we treated H460-CDDP6d and control cells with various doses of Thapsigargin, the well-known ER stress inducer, then monitored the cell viability. While the IC_50_ in wtH460 is 96.57 μM, the IC_50_ in H460-CDDP6d is 45.65 μM, suggesting that the ER compartment might be sensitive for ER stress induced apoptosis (Fig. 5a). Moreover, we observed the comparable sensitivity to cisplatin between wtH460 and H460-CDDP6d in which the IC_50_ was found at 104.78 and 87.62 μM., respectively (Fig. 5b).

To translate this finding to a better clinical relevance, we employed the use of clinically available drugs that target ER homeostasis, Bortezomib (BTZ). The clinical uses of this drug primarily in hematopoietic malignancy, however, the clinical testing are now undergoing to investigate the feasibility of using BTZ in other types of cancers, especially in solid cancers^32,33^ as well as a senolytic drug^34^. We initially treated wtH460 with BTZ and subjected the cells for western blot analysis of GRP78. As shown in Fig. 5g, BTZ treated cells has dramatically increased GRP78 expression, suggesting that BTZ strongly altered the ER homeostasis in H460 cells. Next, we examined the sensitivity of H460-CDDP6d and control cells on BTZ treatment. H460-CDDP6d was found to be more susceptible to BTZ treatment with the IC_50_ 6.84 μM whereas wtH460 has an IC_50_ of 11.20 μM., suggesting the increase in the sensitivity to BTZ in H460 senescence cells (Fig. 5c).

In an array of testing, H460 was found in the group of GRP78Lo during senescence. We then asked whether BTZ may result in a different sensitizing effect on GRP78Hi senescence cells, we then performed the cell viability testing upon BTZ treatment in A549 cells. As expected, we found the IC_50_ of A549-CDDP6d is higher than the wild type cells, suggesting that the increased GRP78 level in A549 during senescence promotes their survival upon ER stress triggered by BTZ in concomitant with another unknown survival pathway against BTZ (Fig. 5d). To further confirm that GRP78 regulates BTZ sensitivity, we treated H460 CRISPR-P131, H460 GRP78-OE. As expected, H460 CRISPR-P131 seems to be more sensitive to BTZ treatment whereas H460 GRP78-OE were more resistant with the IC_50_ of 4.30 and 20.68 μM., respectively (Fig. 5e,f). In order to assess the role of GRP78 in H460-CDDP6d and A549-CDDP6d, the western blot analysis suggested that GRP78 protein is unable to increased upon BTZ treatment in H460-CDDP6d compared to control, which is different from A549 (Fig. 5g). In addition, we also compared the IC_50_ of H460-CDDP6d with the non-tumorigenic lung epithelial cell line, BEAS-2B, upon BTZ treatment. The result suggested that H460-CDDP6d has more susceptible with BTZ than BEAS-2B with the IC_50_ of 6.56 and 15.44 μM., respectively (Supplementary Fig. S7) Taken together, the GRP78 level during senescence protects the cells from BTZ treatment.

As GRP78 has been shown to regulate BTZ resistant senescence cells, we then further test whether the manipulation of GRP78 in senescence cells may reduce cell cycle re-entry in the senescence A549 cells. After treated A549-CDDP6d with IT-139, ruthenium-containing small molecule antitumor drug, the compound exhibits IC_50_ of 247 μM in WT A549 cells (Fig. 5H) with the significant GRP78 reduction can be observed at the dose as low as 6.25 μM (Fig. 5i). We firstly tested whether IT-139 would sensitize A549-CDDP6d to BTZ by pretreating the cells with 12.5 μM IT-139 at 3 h prior to BTZ treatment (Fig. 5j). Pretreating the cells with IT-139 was shown to reduce the IC_50_ from 32.86 to 11.13 μM against BTZ, suggesting IT-139 might be served as BTZ sensitizing agent in senescence cells that express high GRP78 (Fig. 5k). Furthermore, we attest the role of IT-139 as senescence evasion inhibitor by treating the cells after CDDP removal and subsequent monitoring on the cell growth (Fig. 5l). Interestingly, IT-139 abrogated cell cycle re-entry of A549-CDDP6d cells whereas control cells were able to enter cell cycle and proliferate rapidly until day 18 (Fig. 5m). We also demonstrated that IT-139 significantly reduces GRP78 expression in A549 CDDP6d, suggesting that GRP78 is required for BTZ resistance (Fig. 5n). In addition to IT-139 treatment, we have validated the role of GRP78 in A549 senescence cells by transducing the cells with lentiviral particles containing GRP78 shRNA after A549 become senescence. As lentiviral transduction mediates GRP78 knock-down (Supplementary Fig. S8C), it was demonstrated that the reduction of GRP78 in A549-CDDP6d sensitize the cells to Bortezomib treatment (Supplementary Fig. S8A), and attenuates senescence evasion (Supplementary Fig. S8B). To further demonstrate the GRP78 roles in Bortezomib resistance and senescence evasion, we induced H460 and H460 GRP78-OE with CDDP to become senescence, then further compared the Bortezomib IC_50_ as well as proliferation upon CDDP removal. As expected, we found that H460 GRP78-OE-CDDP6d have the IC_50_ of 26.25 μM compared to 8.53 μM in H460 CDDP6d (Fig. 5o). Moreover, H460 GRP78-OE-CDDP6d cells also have higher proliferation upon CDDP removal (Fig. 5p). Finally, in order to confirm that the apoptosis found in H460-CDDP6d and A549-CDDP6d upon BTZ treatment is mediated by ER stress induced apoptosis, we monitored CHOP protein expression as a marker for these phenomena. After normalized and compared with their respective control, H460-CDDP6d has 8-folds CHOP protein expression whereas only twofold in A549 (Fig. 5q,r). This results suggested that BTZ treatment significantly triggers ER stress induced apoptosis, where the GRP78 level is critical to maintain their viability. Taken together, GRP78 contributes to Bortezomib resistance and senescence evasion and the GRP78 reduction by using IT-139 might be served as Bortezomib sensitizing agent as well as senescence evasion inhibitor.

## Discussion

Cisplatin (CDDP) is considered as the mainstay for NSCLC treatment regardless of the stage of cancer^35,36^. Clinically, CDDP has been infused to the patients in every 3-week cycle with at least 3–4 cycles to justify its efficacy. This repeated regimen is associated with CDDP retention, especially in the liver. In some cases, the retention of cisplatin can be detected even the patients have stopped using the drug for a decade^37–40^. This may lead to the concern of potential long-term toxicity. In addition to those concerns, the retention of CDDP at sub-toxic doses may potentially induce cellular senescence based on our results as well as others^41^. Indeed, intrinsic resistance to CDDP has been reported in which the proposed mechanisms include the downregulation of the plasma membrane transporter, CTR1, resulting in reduced CDDP uptake in CDDP resistant cells. Moreover, the post-target resistance predominantly involved with TP53 mutation as wild-type TP53 reportedly has a greater benefit from CDDP-based chemotherapy than in the patients with TP53 mutations^42^. CDDP-induced senescence provides an additional mechanism, in which the tetra ploidy characteristic found in senescence promotes the stoichiometric process that the double amount of DNA accounts for the resistance^43,44^. In addition, the senescence cell manifested an increase in pro-inflammatory cytokines production, as characterized by SASP. Our finding suggested that cytokines such as IL-1α, IL-1β has been transcriptionally increased in which it may subsequently be secreted and promote an inflamed tumor microenvironment^45^. We have found that the conditioned medium taken from H460-CDDP6d promotes wtH460 cell proliferation significantly, which is, in contrast to A549-CDDP6d (Supplementary Fig. S1). This suggested that the level and the type of cytokines released from these different senescence cells have a varying degree of tumor promotion. Overall, senescence is not merely promoting CDDP resistance but also promotes the resurgence of the subpopulation with altered properties, culminating in the subsequent relapse.

Senescence is one of the stress responses that halts damaged or stressed cells. Indeed, it was found that the stress responses such as unfolded protein response (UPR) may occur during senescence cell fate^11^. Given that the SASP phenotype is one of the key characteristics of senescence cells, this increases the rationale to further characterize the UPR mechanism during senescence. Several lines of evidence indicate that ER stress occurs in the correlation with senescence regardless of senescence inducers including replicative, oncogenic activation, DNA-damaging agents, or oxidative stress^46–49^. One of the key signatures of ER stress includes GRP78 mRNA and protein expression was shown to be altered during senescence. As GRP78 is considered the master regulator of ER stress, its role in senescence however remains to be obscured since its level was found to be increased^50,51^ or decreased^48,49^, depending on cellular context. Our study indicates that GRP78 protein levels may differentially be expressed during senescence, as H460-CDDP6d exhibits a downregulation of GRP78 whereas GRP78 in A549-CDDP6d are stable. As in Fig. 3b,c, GRP78 protein levels are reduced where its transcripts were found slightly increased. This phenomenon can be explained by the fact that GRP78 protein has a tightly translational control during stress conditions in which translation efficiency is enhanced^22^. We found that GRP78 mRNA transcripts during senescence in H460 are around 2.5-fold whereas more than a tenfold increase in A549 (Fig. 3 and Supplementary Fig. S1), this suggested that A549 equipped with better molecular machinery to cope with the stress where it can produce more GRP78 transcripts. In H460, however, the level of GRP78 transcripts seems not enough to promote sufficient GRP78 expression and this inferior capacity leads them to be more susceptible to additional ER stress that may occur from the following treatment. We further elucidated that GRP78 level in senescence cells are responsible for cell cycle re-entry, interestingly we found that GRP78 expression during senescence promotes senescence evasion. As GRP78 translocates from the ER lumen to the surface of tumor cells, the oncogenic activity of GRP78 was found to be partly contributed by the phosphoinositide 3-kinase (PI3K) pathway. It was found that anti-GRP78 monoclonal antibody (Mab159) attenuates PI3K signaling and reduces tumor growth and metastasis^52^. Then, we postulated that GRP78 is an upstream activator of the PI3K/Akt pathway, in which the increased GRP78 levels during senescence may promote cell cycle re-entry via PI3K/Akt. However, the actual pathway underlying the GRP78 function that promotes cell cycle re-entry still needs further investigation.

GRP78 localized in the plasma membrane as well as getting secreted. Some of the evidence demonstrated that GRP78 level in plasma may correlate with the disease stage, as plasma GRP78 was found higher in late-stage non-small cell lung cancer patients compared to an early stage^53^, then GRP78 might be one of a good candidate for a novel prognostic marker for lung cancer. As in our study it found the relationship of GRP78 expression during senescence and reversible senescence, and the GRP78 plasma level might be a good predictor of tumor recurrence, however, additional studies may be required to ascertain this possibility.

The level of GRP78 not only promotes reversible senescence, as our finding also suggested that GRP78 contributes to the resistance to therapy. GRP78 overexpression is associated with chemo resistances such as Doxorubicin and other targeted therapy^54^. Senescence cells also exhibit intrinsic drug resistance by nature, the search for the potential compound targeting senescence cells is in high demand. This group of agents has been classified as senolytic drugs, in which they are targeting molecular targets that are critical for senescence cell survival. There have been classified senolytic drugs for specific targets such as Bcl-2 and other targets^55^. Among these senolytic agents, Bortezomib has gained a lot of attention as it might potentially be used as a senolytic drug^34^. Since the mechanism of Bortezomib is enhanced ER stress from proteasomal inhibition, we speculated that this drug may enhance more ER stress in the senescence cells in which have pre-existing ER stress. Interestingly, in the senescence cells with GRP78 downregulation, these cells were more susceptible to Bortezomib treatment. On the contrary, in the increase GRP78 level in A549-CDDP6d, this senescence cells appear to be less susceptible to Bortezomib than in wild type cells (Fig. 5i). The GRP78 reduction by IT-139, the ruthenium-containing small molecule antitumor drug that has been previously report on its activity in GRP78^56^, was shown to enhance BTZ activity and reduce senescence evasion in this study. As IT-139 was shown to have a good safety profile, either preclinical or clinical testing on its role as senescence evasion inhibitor and BTZ sensitizing agent is deemed possible.

Indeed, apart from our interest in GRP78 as a critical regulator of the reversible senescence and the resistant to treatment, we have identified that the activation of IRE-1α, manifested by increasing p-IRE-1α expression without CHOP activation, is occurred in H460-CDDP6d. This suggested that subtle ER stress activation occurs in these senescence cells, however, this ER stress was not accompanied by XBP-1 mRNA splicing. This scenario seems similar to the HRAS induces senescence, where the hyperactivation of IRE-1α regulates senescence without XBP-1 activities^57^. Further investigation on CDDP induces senescence via IRE-1α regulation is also warranted.

## Materials and methods

### Cell lines

The human non-small cell lung cancer cells (NSCLC) including H460, H23, H292 and A549 cell line was obtained from the American Type Culture Collection (ATCC, Manassas, VA, USA). All cell lines except A549 were cultured in Roswell Park Memorial Institute (RPMI) 1640 medium supplemented with 10% fetal bovine serum (FBS), 2 mmol/L L-glutamine and 100 units/mL penicillin/ streptomycin (Gibco, Gaithersburg, MA, USA) in a 5% CO2 at 37 °C. The A549 cells was maintained in Dulbecco's Modified Eagle Medium (DMEM, high glucose medium) supplemented with 10% fetal bovine serum (FBS), 2 mmol/L L-glutamine and 100 units/mL penicillin/ streptomycin (Gibco, Gaithersburg, MA, USA) in a 5% CO_2_ at 37 °C. All NSCLCs cell lines were incubated to 70–80% confluence for further experiments.

### Patient-derived primary lung cancer cell line preparation from malignant pleural effusion

The patient-derived malignant cancer cells were isolated from pleural effusions of recurrent or advanced stage non-small cell lung cancer patients who had been diagnosed at the King Chulalongkorn Memorial Hospital. The protocol of conduction was approved by the Ethics Committee of the Faculty of Medicine, Chulalongkorn University, Bangkok, Thailand (IRB 365/62) and was obtained informed consents from all participants. This study was carried out in accordance with the principles of the World Medical Association Declaration of Helsinki. Primary cancer cells were collected from pleural effusion (500–1000 mL) through thoracentesis. The collected samples were centrifuged at 300 g for 10 min, at 4 °C and the cells were resuspended in RPMI medium with 10% FBS, 2 mM L-glutamine, and 100 units/mL of each of penicillin and streptomycin. After culturing for 10–15 passages, they were characterized as the patient-derived primary cancer cell lines (ELC16, ELC20). The characteristics as well as their status of mutation were described previously^58^.

### Reagents

The 3-(4, 5-Dimethylthiazol-2-yl)-2, 5-diphenyltetrazolium bromide (MTT). Propidium iodide (PI) were supplied from Sigma-Aldrich Corporation (St. Louis, MO, USA). The RPMI 1640, DMEM, phosphate-buffered saline (PBS) pH 7.4, trypsin, L-glutamine, fetal bovine serum (FBS) and penicillin/streptomycin solution were bought from Gibco (Gaithersburg, MA). Agarose was supplied from Bio-Rad (Hercules, CA, USA). Clarity and Clarity Max Western ECL Substrates (cat: 1705060) is obtained from Bio-Rad. Pierce BCA Protein Assay Kit were obtained from Thermo Fisher Scientific (Rockford, IL, USA). CDDP (Pt(NH3)2Cl2) was purchased from Sigma-Aldrich Corporation. Velcade (Bortezomib) was kindly provided by Dr. Sudjit Luanpitpong, Mahidol University, Bangkok, Thailand. The G418 sulfate, Neomycin (50 mg/mL) was obtained from Thermo Fisher Scientific. The IT139, the GRP78 inhibitor was supplied from Adooq Bioscience, USA. The senescence β-galactosidase staining kit (cat no: #9860) and primary antibodies kit for ER stress (cat no: #9956) was purchased from cell signaling Technology (USA). The cellular senescence marker and secretory phenotype PCR primers were obtained from Macrogen, Korea.

### Investigation for IC_50_ value

The H460, H23, H292, A549, ELC16 and ELC20 cells (1 × 10^4^ cells/well) were seeded into 96-well plates. Then various concentrations of CDDP (2.5–160 µM) or Bortezomib were treated to the cells. After 24-h incubation, MTT (3-(4, 5-dimethylthiazol-2-yl)-2,5-diphenyltetrazolium bromide) (5 mg/mL) reagent was dissolved in medium and incubated for 3–4 h. Subsequently, the MTT reagent was discarded and 100 µL/well of DMSO was added to each well to dissolve the crystals. The absorbance was measured at a wavelength of 570 nm by using a microplate reader. The IC_50_ values were determined at the dose that exhibit 50% cell viability compared with the untreated controls. The IC_50_ values were determined by using Excel software (Microsoft).

### Proliferation assay

For cell proliferation assay, initial cell density was 1.5 × 10^3^ cells/well. All NSCLC cells were treated with CDDP (0, 1, 2.5, 5, 10, 20 µM). The cell proliferation was monitored from day 1 to day 6 with MTT. The proliferation rate was calculated by percent viability = (Average A_570_ CDDP/Average A_570_ control) × 100.

### Clonogenic assay

The H460 cells and A549 cells were seeded in 24 well plate at cell density 7.5 × 10^3^ cell/well for 24 h for cell adhesion, then incubated with complete medium containing CDDP at various concentration (0, 1, 2.5, 5, 10, 20 µM) for 6 days. After that, the cells were trypsinized and reseeded in CDDP-free complete medium for further 7 days incubation. The observed colonies were washed with PBS, fixed with methanol: acetic acid (3:1) for 5 min, then stained with 0.05% w/v crystal violet in 4% formaldehyde for 30 min. Finally, the samples were washed with water and counted the number of stained colonies compared to control.

### Cell cycle analysis

For cell cycle analysis, the cells were suspended in 1 mL PBS. The suspended cells were centrifuge at 450xg, 5 min, 25 °C and fixed the cells for 24 h in 1 ml of 70% ethanol. After fixation, the suspended cells were centrifuge at 450xg, 5 min, 25 °C and washed with PBS (1 mL). The cells were resuspended in 300 µl PI/Triton X-100 staining solution and incubated at 37 °C for 30 min then subjected for flow cytometry analysis. The results were analyzed via FlowJo software.

### Senescence β-galactosidase cell staining

The senescence β-Galactosidase levels were detected by using the β-Galactosidase staining kit (cat no: #9860) from cell signaling. The cells were seeded in 96 well/plate for cell density at 1 × 10^4^ cell/well overnight. The following day, the fixative solution (1x) was placed to each well and incubated for 15 min at room temperature. After fixation, the cells were washed with 2xPBS. The β-Galactosidase Staining Solution was added to the cells for overnight incubation at 37 °C. The senescence cells were developed blue colour and the pictures were taken at 10 × magnification. For quantification of positive cell staining, the 5 random fields were selected and counted the total cell versus β-Galactosidase positive cells.

### Real time quantitative PCR

The total RNA from CDDP induced senescence cells (1 × 10^5^ cells) were extracted with TRIzol reagent. The total RNA was reverse transcribed using SuperScript III reverse transcriptase (Invitrogen), according to manufacturer’s instruction. RT-qPCR was performed in quadruplicate with exactly 100 ng of total RNA using QuantiFast SYBR Green PCR Master Mix (Qiagen, Applied Biosystems, Life Technologies Inc.) in the ABI Step One Plus Real-Time PCR system with the following primer sets (Macrogen, Korea).

**Table:** 

List of primers	
Primer	Sequences (Tm)
GRP78-fwd	GTTCTTCAATGGCAAGGAACCATC (63.5 °C)
GRP78-rev	CCATCCTTTCGATTTCTTCAGGTG (63.5 °C)
p53-fwd	CTTCCTGCAGTCTGGGACAGC (65.3 °C)
p53-rev	GCAGCTGGGCCTACAGCACACG (69.6 °C)
p21-fwd	TCTTGCACTCTGGTGTCTGA (58.4 °C)
p21-rev	CTGCGCTTGGAGTGATAGAA (58.4 °C)
p16-fwd	CGTACCCCGATTCAGGTG (58.4 °C)
p16-rev	ACCAGCGTGTCCAGGAAG (58.4 °C)
p57-fwd	GCGGTGAGCCAATTTAGAGC (60.5 °C)
p57-rev	CGGTTGCTGCTACATGAACG (60.5 °C)
IL-1α-fwd	GTAAGCTATGGCCCACTCCA (60.5 °C)
IL-1α-rev	AGGTGCTGACCTAGGCTTGA (60.5 °C)
IL-1β-fwd	CTGAAAGCTCTCCACCTC (56.1 °C)
IL-1β-rev	GATCTACACTCTCCAGCTG (57.3 °C)
IL7-fwd	CTCCAGTTGCGGTCATCATG (60.5 °C)
IL7-rev	GAGGAAGTCCAAAGATATACCTAAAAGAA (64.8 °C)
IL8-fwd	CTTTCCACCCCAAATTTATCAAAG (60.1 °C)
IL8-rev	CAGACAGAGCTCTCTTCCATCAGA (65.3 °C)
CSF2 (GM-CSF)-fwd	GGCCCCTTGACCATGATG (58.4 °C)
CSF2 (GM-CSF)-rev	TCTGGGTTGCACAGGAAGTTT (59.4 °C)
CXCL1-fwd	GAAAGCTTGCCTCAATCCTG (58.4 °C)
CXCL1-rev	CACCAGTGAGCTTCCTCCTC (62.5 °C)
CXCL2-fwd	AACTGCGCTGCCAGTGCT (58.4 °C)
CXCL2-rev	CCCATTCTTGAGTGTGGCTA (58.4 °C)
GAPDH-fwd	CCACCCATGGCAAATTCCATGGCA (67 °C)
GAPDH-rev	TCTAGACGGCAGGTCAGGTCCACC (70.4 °C)
XBP1-Fwd	AAACAGAGTAGCAGCTCAGACTGC (65.3 °C)
XBP1-Rev	TCCTTCTGGGTAGACCTCTGGGAG (68.7 °C)
CHOP-fwd	AGTGCCACGGAGAAAGCTAA (56.3 °C)
CHOP-rev	CCATACAGCAGCCTGAGTGA (56.5 °C)
ATF6-fwd	GCCTTTATTGCTTCCAGCAG (54.5 °C)
ATF6-rev	TGAGACAGCAAAACCGTCTG (55.5 °C)
E-cadhein-Fwd	TTAAACTCCTGGCCTCAAGCAATC (61 °C)
E-cadhein-Rev	TCCTATCTTGGGCAAAGCAACTG (60.6 °C)
N-cadhein-Fwd	GACCGAGAATCACCAAATGTG (57.9 °C)
N-cadhein-Rev	GCGTTCCTGTTCCACTCATAG (59.8 °C)
Vimentin-Fwd	ACCCTGCAATCTTTCAGACAG (57.9 °C)
Vimentin-Rev	GATTCCACTTTGCGTTCAAGG (57.9 °C)
Slug-Fwd	AGCATTTCAACGCCTCCA (53.7 °C)
Slug-Rev	GGATCTCTGGTTGTGGTATGAC (60.3 °C)

### *XBP1* splicing assay by PCR

*XBP1* splicing assay was conducted to confirm the IRE1α activation. Briefly, first-strand cDNA was synthesized from the total RNA isolated from CDDP6d and control cells using SuperScript III reverse transcriptase (Invitrogen). Since activated IRE1α cleaves the *XBP1* mRNA (*XBP1u*, 473 bp) to a shorter *XBP1* mRNA that lacks a 26 bp fragment, resulting in the loss of *Pst*I restriction site (*XBP1s*, 447 bp). cDNA from the spliced form *XBP1s* is resistant to *Pst*I, but *XBP1u* is digested by *Pst*I into 290 and 183 bp fragments. The cDNAs of *XBP1u* and *XBP1s* were amplified by PCR using the same primer set: 5′-AAACAGAGTAGCAGCGCAGACTGC-3′ and 5′-TCCTTCTGGGTAGACCTCTGGGAG-3′. The amplicons were digested by *Pst*I, resolved on 2% agarose gels containing ethidium bromide, and then visualized using the Gel Doc XR + system (Bio-Rad). The 447 bp band represented the cDNA amplicon from the processed *XBP1s*, whereas both the 290 and 183 bp bands originated from the cDNA amplicon from the unprocessed *XBP1u*.

### Colony formation assay (anchorage-independent growth)

The colony formation assay was measured the single cell survival rate in anchorage-independent growth. The colony formation assay contains 2 layers of agarose with different composition. In lower layer, the 1% agarose was mixed with 2 × complete medium (1:1) ratio. The mixture 1.5 mL/well was placed in 6 well plate. The upper layer was contained the mixture of agarose (0.6%) and single cells suspension of cells and CDDP induced senescence cells (cell density 7500 cells/well) and 2 complete medium (1:1) ratio (1.5 mL/well). After setting the upper layer, the plates was incubated at 5% CO_2_ for 37 °C. Fresh complete 2 × complete medium (100 µl/well) was added every 3 days to prevent dryness the cell surface layer. The colony formation was taken the photo at day 0, 7 and 14 days. The colony formation was analyzed by using image J software.

### Western blot analysis

The CDDP induced senescence cells was lysed with lysis buffer (cat no: #9803, cell signaling) with complete protease inhibitor cocktail tablets provided in EASYpack (Roche, cat no: 04693116001)]. The supernatant was collected and determined for protein content according to the protocol by using the Bicinchoninic acid (BCA) protein kit (Thermo-Fisher scientific, Rockford, USA). The 30 µg protein was loaded on 10% sodium dodecyl sulfate polyacrylamide gel electrophoresis (SDS-PAGE) and run with running buffer. After separation by SDS-PAGE, the protein was transferred onto 0.45 µm nitrocellulose membrane by using semi-dry transfer method. The transferred membrane was blocked with 5% nonfat milk in TBST (25 mM Tris–HCl (pH 7.5), 125 mM NaCl, 0.05% Tween 20) for about 1 h and incubated with primary antibodies rabbit GRP78 (78 kDa, 1:1000 in 5% w/v BSA in TBST, cell signaling, cat no:3177) and ER stress markers such as rabbit PERK (140 kDa, 1:1000 in 5% w/v BSA in TBST, cell signaling, cat no:5683), rabbit IRE1α (130 kDa, 1:1000 in 5% w/v BSA in TBST, cell signaling, cat no:3294), rabbit pIRE1α (phosphor S724) (110 kDa, 1:1000 in 5% w/v BSA in TBST, abcam, ab48187), rabbit PDI (57 kDa, 1:1000 in 5% w/v BSA in TBST, cell signaling, cat no:3501), rabbit Ero1-Lα (60 kDa, 1:1000 in 5% w/v BSA in TBST, cell signaling, cat no:3264), mouse CHOP (27 kDa, 1:1000 in 5% w/v BSA in TBST, cell signaling, cat no:2895), rabbit ATF-6 (D4Z8V) (100 kDa, 1:1000 in 5% w/v BSA in TBST, cell signaling, cat no:65880) for overnight at 4 °C. Rabbit calnexin (90 kDa, 1:1000 in 5% w/v BSA in TBST, cell signaling, cat no: 2679), Rabbit β-actin (45 kDa, 1:1000 in 5% w/v BSA in TBST, cell signaling, cat no: 4967) and Rabbit GAPDH (37 kDa, 1:1000 in 5% w/v BSA in TBST, cell signaling, cat no: 2118) were used as loading control. The membrane was incubated with horseradish peroxidase (HRP) conjugated with secondary antibodies, anti-rabbit IgG, HRP-linked antibody (5% w/v skim milk in TBST, cell signaling, cat no: 7074) and anti-mouse IgG, HRP-linked antibody (5% w/v skim milk in TBST, cell signaling, cat no: 7076) for 1 h at room temperature. The complex reactivity was detected by using with chemiluminescence substrate. The protein expression level was calculated by image J software.

### Genome engineering mediated knockdown GRP78 by clustered regularly interspaced short palindromic repeats (CRISPR)/ CRISPR-associated protein 9 (Cas9)

The Cas9 nuclease was targeted by 20 nucleotides guide sequence within the single guide RNA. The targeted sequences for sgRNA are in Exon 3 in the GRP78 gene. The design of sgRNA was.

**Table:** 

Oligo Name	5′-Oligo Seq-3′	Oligo length (bp)
GRP78-131-Fwd	CAC CGC CAT ACA TTC AAG TTG ATA	24
GRP78-131-Rev	AAA CTA TCA ACT TGA ATG TAT GGC	24

The H460 cells (1 × 10^5^ cell/well) were seeded in 24 well plate and transfected with GRP78 CRISPR/Cas9 knockdown plasmids by using Lipofectamine 2000 reagent (Invitrogen, Carlsbad, USA) for 48 h. The transfected cells were selected by puromycin and single clone isolation method. After expanded H460 knockdown GRP78 cells population was confirmed by western blot analysis using anti-GRP78 antibodies.

### The generation of GRP78 overexpressing H460 cell lines

The H460 cells (1 × 10^5^ cell/well) were seeded in 24 well plate and transfected with pc.DNA3.1(+)-GRP78/BiP plasmids (Addgene, Plasmid #32701) by using Lipofectamine 2000 reagent (Invitrogen, Carlsbad, USA) for 48 h. The overexpression cells population were selected by treating with G418 (1.6 mg/mL) 72 h after transfection. The survival cell population were grown in 6 well plate with complete RPMI medium and screened by western blot analysis using anti-GRP78 antibodies.

### The generation of GRP78 knock-down in A549-CDDP6d by shRNA lentiviral particles transduction

The senescence A549 with GRP78 reduction were generated according to manufacturer instructions (Santa Cruz Biotechnology [Cat. No. sc-29338-V]). Briefly, the GRP78 shRNA (h) lentiviral particles at 1.0 × 10^5^ infectious units of virus (IFU), which containing 3 target-specific constructs encoding 19–25 nt (plus hairpin) shRNA designed to knock-down GRP78 gene expression, were added to the A549-CDDP6d cells and incubated overnight. Afterwhich, the cells were selected for stable clones for subsequent experiments and the level of GRP78 was confirmed by western blot.

### Statistical analysis

All experiments were performed three independent analysis. The degree of the spread of data was expressed by the standard deviation (± s.d.). Two-tailed Student’s t-test was used to compare the means of two groups. The statistical significance was evaluated with p-value less than 0.05.

## Conclusions

Taken together, this is the first report that highlights the role of GRP78 in the reversible senescence as well as the resistance to the therapy targeted proteasomal inhibition by Bortezomib in NSCLCs. Since GRP78 can be measured in the plasma, further evidence on using plasma GRP78 as a prognostic marker for NSCLC progression as well as the predictive response to Bortezomib treatment could potentially be explored.

## Supplementary Information

Supplementary Information.
