# Supplementary material for: GRP78/BiP determines senescence evasion cell fate after cisplatin-based chemotherapy
Source: Sci Rep. 2021 Nov 17;11:22448. doi: 10.1038/s41598-021-01540-8 (PMC8599848; doi:10.1038/s41598-021-01540-8)

**Supplementary Information**

GRP78/BiP determines senescence evasion cell fate after cisplatin-based chemotherapy

Zin Zin Ei, Kanuengnit Choochuay, Alisa Tubsuwan, Decha Pinkaew, Maneewan Suksomtip, Chanida Vinayanuwattikun, Pithi Chanvorachote and Preedakorn Chunhacha


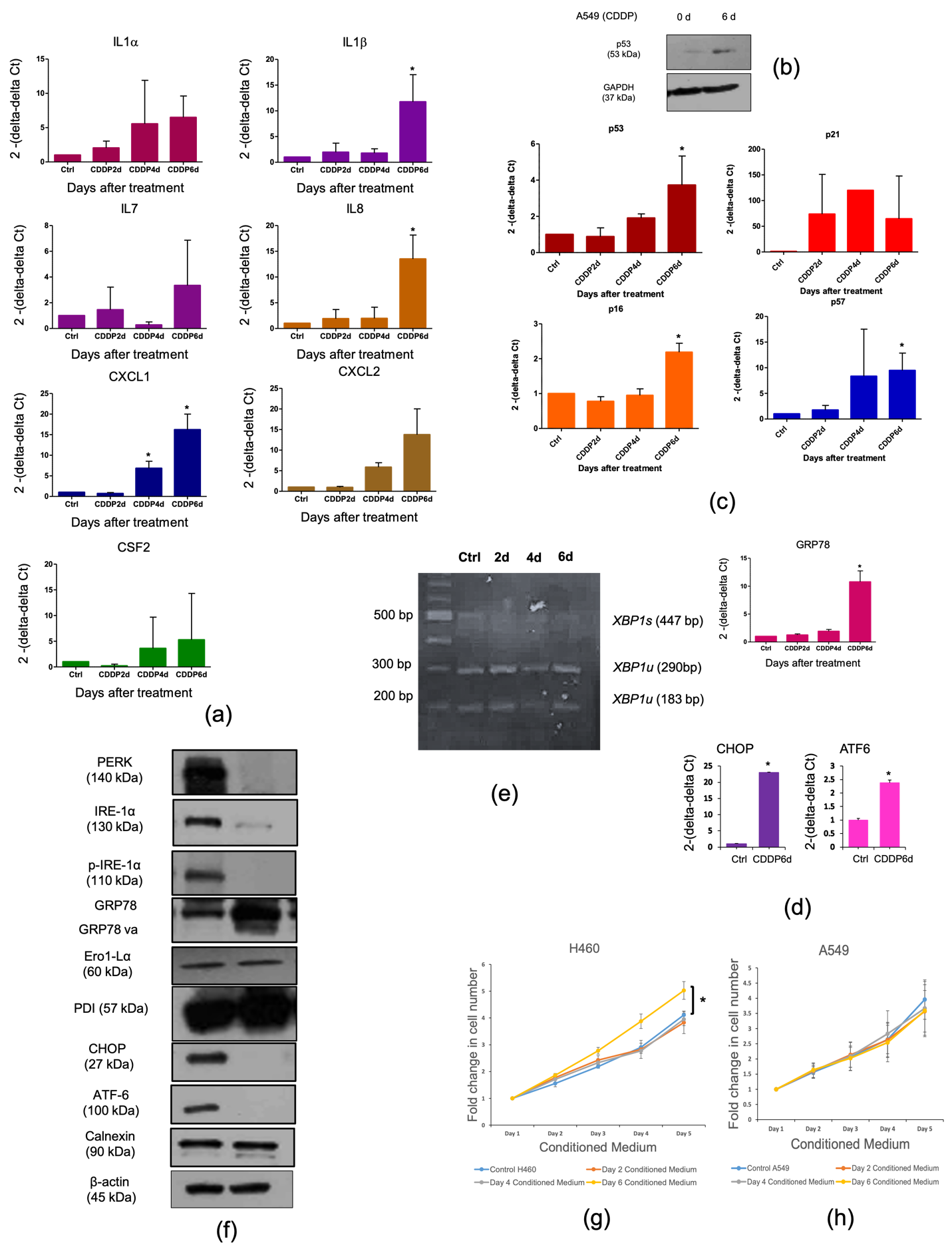


**Supplementary Fig. 1.** **A549 senescence and the UPR analysis**. (**a**) Time-course analysis of the relative mRNA levels of various SASP genes by real-time Q-PCR in A549 cells treated with CDDP at 5 µM. (**b**) p53 Western blot analysis of CDDP induced A549 senescence cells (**c**) Time-course analysis of the relative mRNA levels of p53, p21, p16 and p57 by real-time Q-PCR in A549 cells treated with CDDP at 5µM (**d**) Time-course analysis of the relative mRNA levels of GRP78, CHOP and ATF-6 by real-time Q-PCR in A549 cells treated with CDDP at 5µM (**e**) A549 cells were treated with 5 µM CDDP at the respective days indicated. The cDNA from total RNA from these cells was subjected to PCR and *Pst1* restriction digestion (CTGCA|G) to semi-quantitatively evaluate the levels of *XBP1s*. *XBP1u* unspliced *XBP1*, *XBP1s* spliced *XBP1* (**f**) Protein expression levels of ER stress markers were detected in CDDP induced A549 senescence cells during SASP by western blot analysis (**g**) Conditioned medium from H460-CDDP6d were used to culture wild type H460 cells. (**h**) Conditioned medium from A549-CDDP6d were used to culture wild type A549 cells. For all the data, the average of three independent experiments is shown. Data were analyzed by two-tailed Student’s t-test. *Denotes p < 0.05.


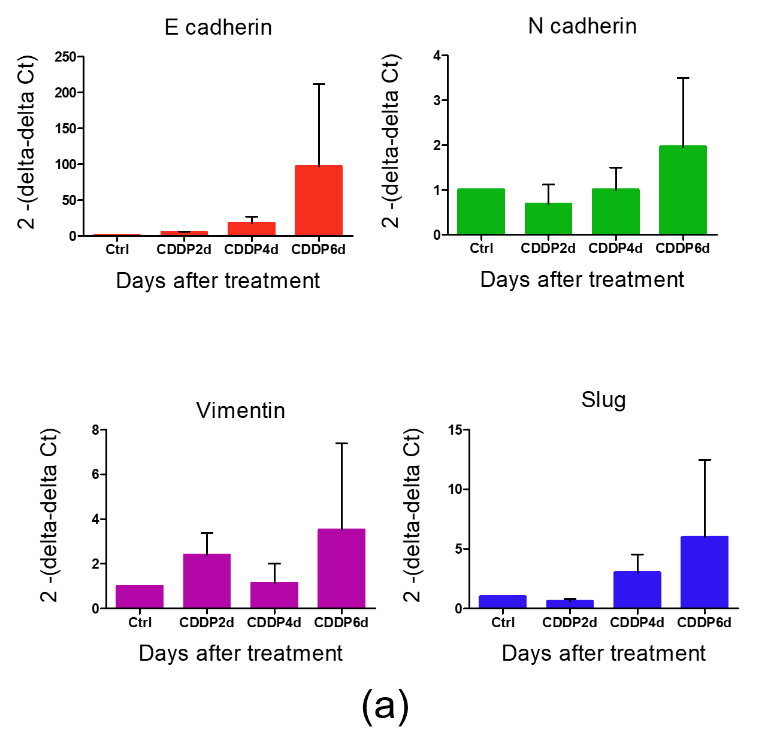


**Supplementary Fig. 2.** **EMT markers in the senescence H460 cells**. EMT markers in H460-CDDP 6d. (**a**) Relative mRNA levels of E-cadherin, N-cadherin, Vimentin and slug in H460-CDDP6d by real time Q-PCR (n=3).


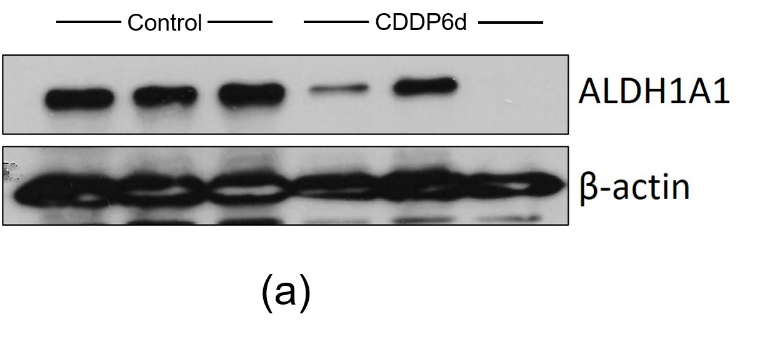


**Supplementary Fig. 3.** **Stem cells marker in H460-CDDP6d**. **(a)** ALDH1A1 protein expression level in CDDP6d (n=3).


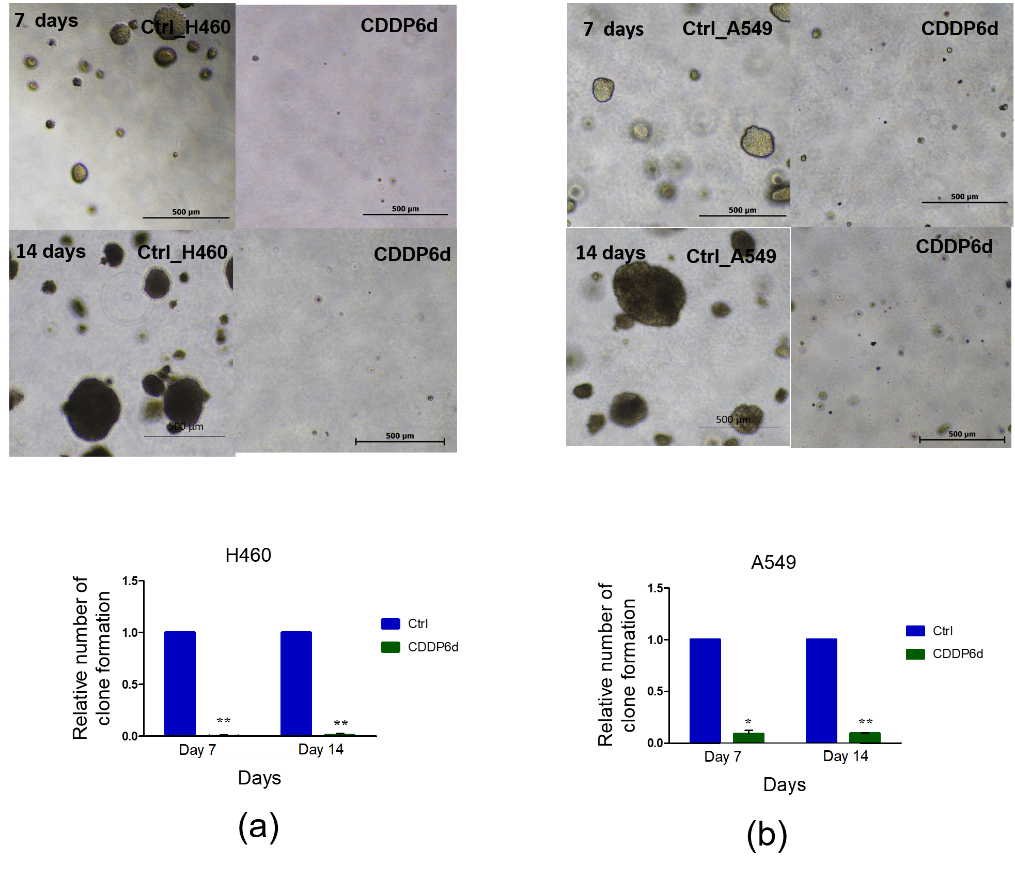


**Supplementary Fig. 4.** **Soft Agar colony formation of H460-CDDP6d and A549-CDDP6d**. Anchorage independent growth assay in H460-CDDP6d (**a**) and A549-CDDP6d (**b**) at day 7 and day 14 showing the number of colonies compared to control. For all the data, the average of three independent experiments is shown. Data was analyzed by two-tailed Student’s t-test. *Denotes p < 0.05, ** denotes p < 0.01. The scale bar is 500 µm.


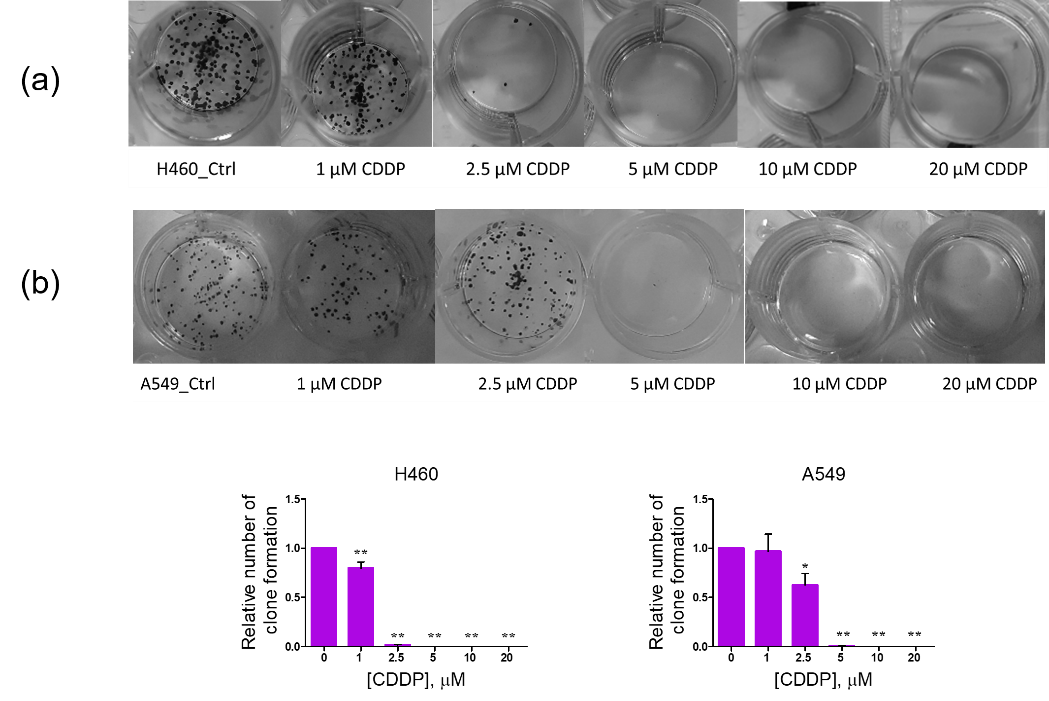


**Supplementary Fig. 5.** **Clonogenic assay of CDDP treated H460 and A549 cells**. H460 cells (a) and A549 cells (b) were treated with various doses of CDDP (1-20 μM) for 6 days then cultured in CDDP free medium for another 6 days. The colonies formed were stained with crystal violet and the ratio were calculated compared to control (n=3). For all the data, the average of three independent experiments is shown. Data was analyzed by two-tailed Student’s t-test. *Denotes p < 0.05, ** denotes p < 0.01.


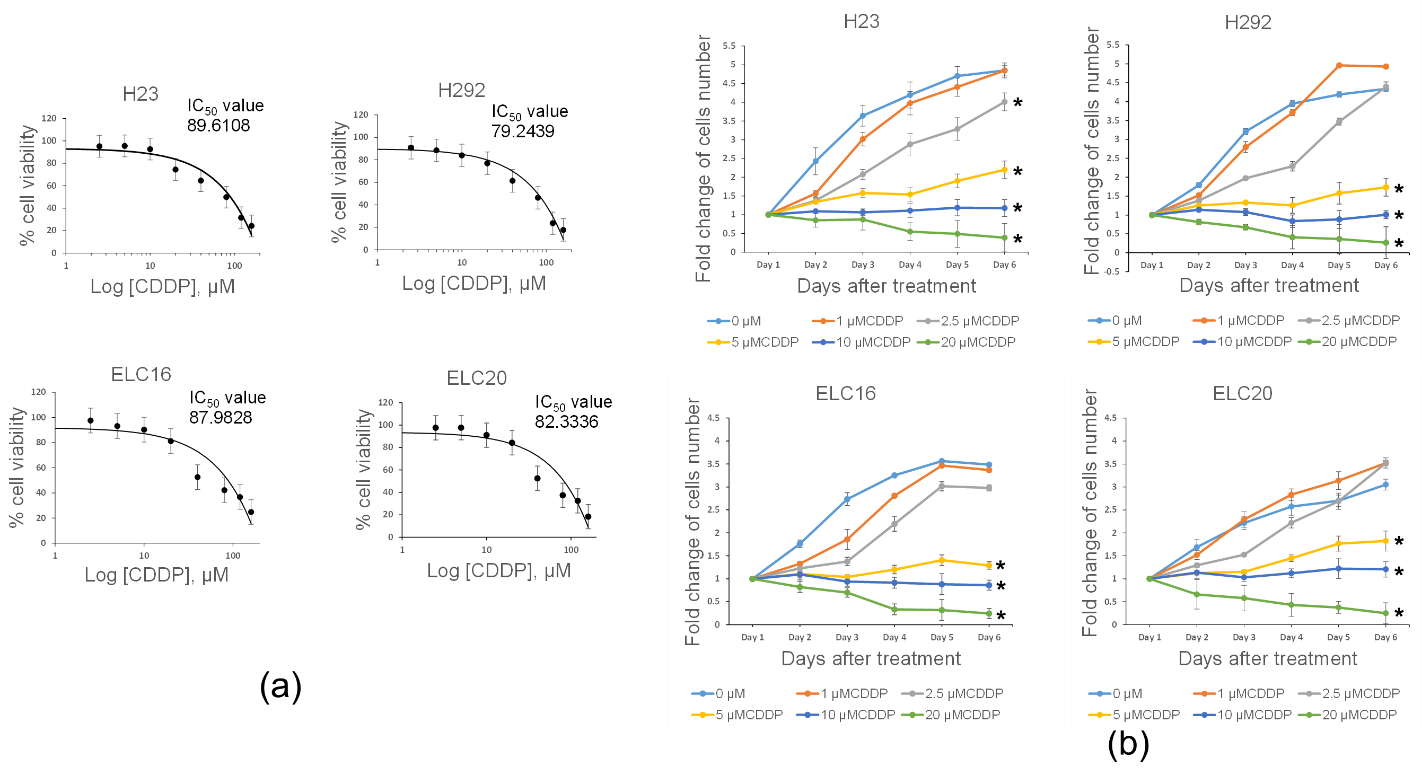


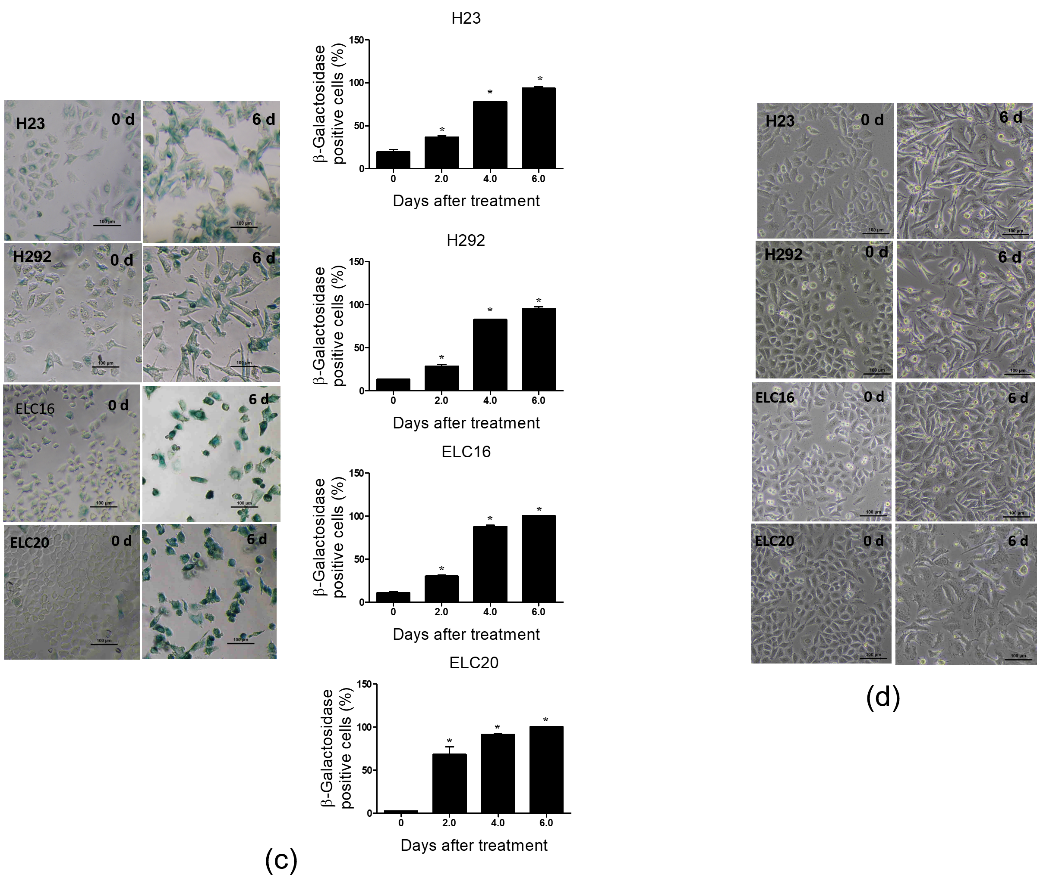


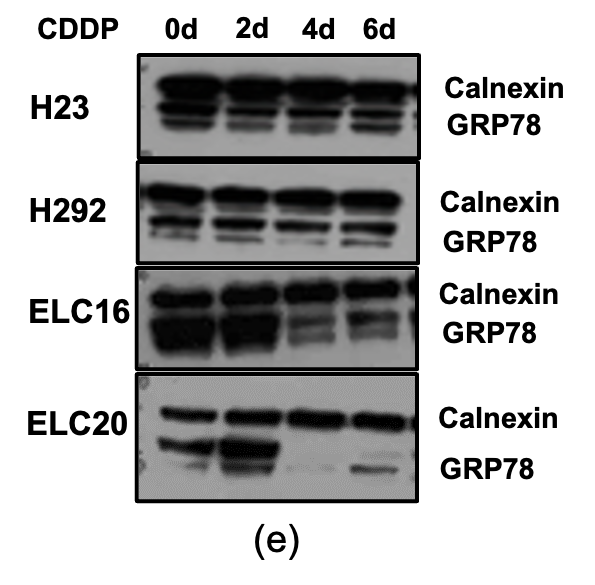


**Supplementary Fig. 6.** **Differential GRP78 protein expression after CDDP induces senescence in H23, H292 and patient-derived Primary Lung Cancer Cell Line (ELC16, ELC20)** (**a**) IC50 values of H23, H292, ELC16 and ELC20 to CDDP. (**b**) Cell proliferation curves of H23, H292, ELC16 and ELC20 exposed to CDDP (1-20 µM) for 6 days. (initial cell concentration is 1.5x10^3^ cells/well) (**c**) Time-course analysis of senescence-associated β-galactosidase in H23, H292, ELC16 and ELC20 cells treated with CDDP at 5µM, the percentage of β-gal blue staining positive cells were calculated (n=3). (**d**) NSCLCs morphology analyzed following 6 days of treatment with CDDP at 5 µM. (**e**) Time-course analysis of the GRP78 protein expression in H23, H292, ELC16 and ELC20 cells treated with CDDP at 5µM. For all the data, the average of three independent experiments is shown. Data was analyzed by two-tailed Student’s t-test. *Denotes p < 0.05. The scale bar in figure (c) and (d) is 100 µm.


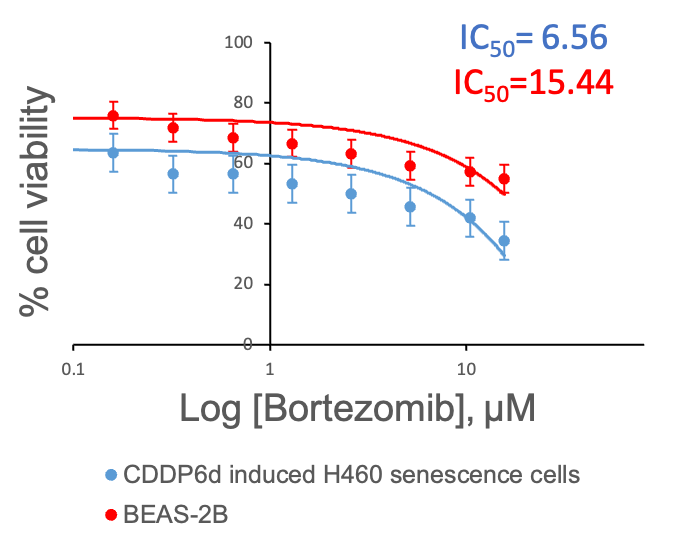


**Supplementary Fig. 7.** **Differential response of the senescence H460 versus non-tumorigenic lung epithelial cell line BEAS-2B.** IC_50_ value for H460-CDDP6d cells and BEAS-2B treated with different concentration of Bortezomib (0.16-15.62 µM).


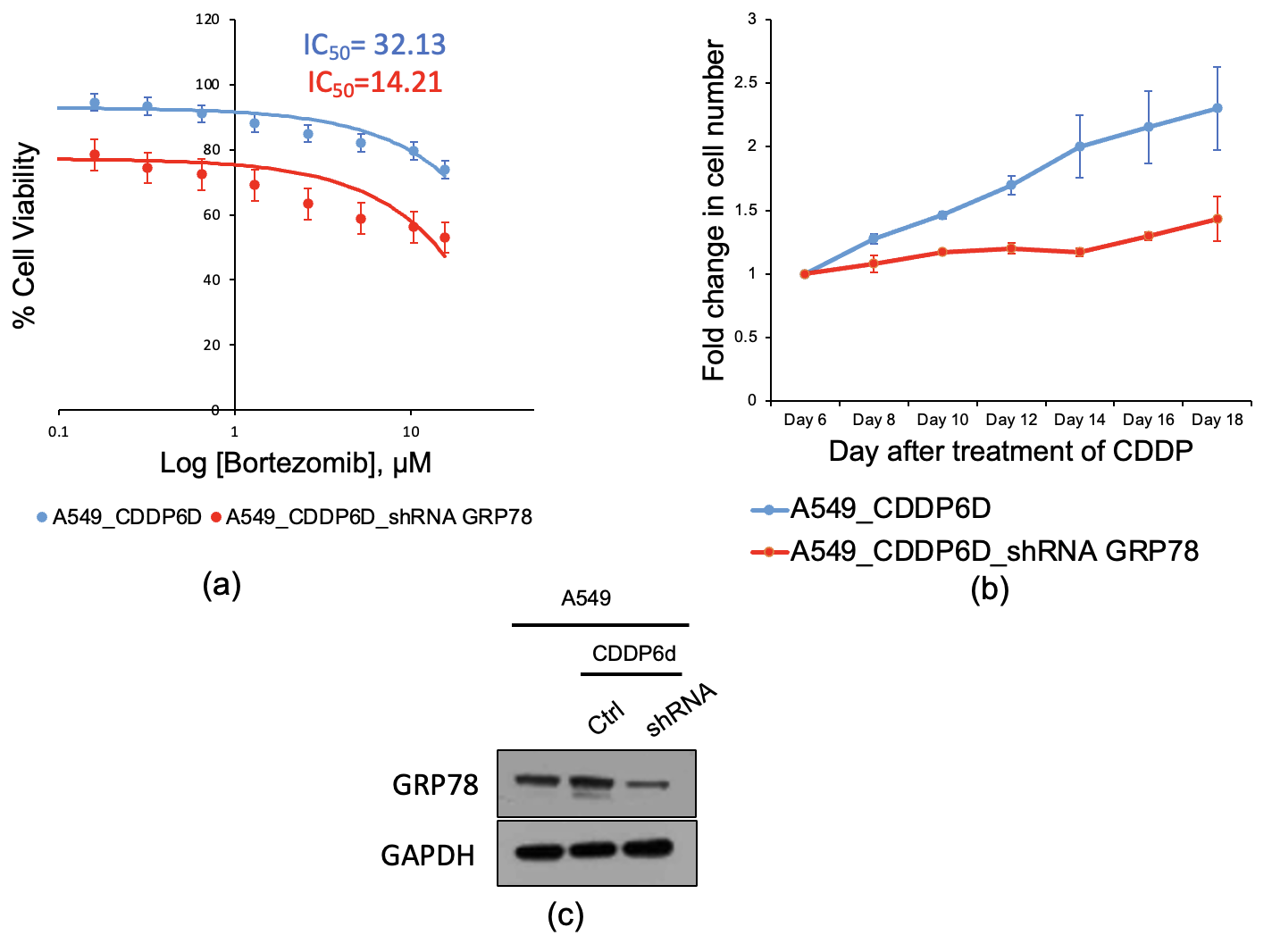


**Supplementary Fig. 8.** **Lentiviral shRNA mediates GRP78 silencing attenuates senescence evasion in A549-CDDP6d**. (**a**) IC_50_ value of Bortezomib treated in A549-CDDP6d and A549-CDDP6d transfected with lentiviral shRNA targeting GRP78. (**b**) Cell proliferation after CDDP removal of A549-CDDP6d versus A549-CDDP6d transfected with lentiviral shRNA targeting GRP78. (**c**) The level of GRP78 protein expression in WT A549, A549-CDDP6d and A549-CDDP6d transfected with lentiviral shRNA targeting GRP78.

***Uncropped gels***


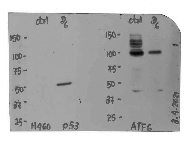

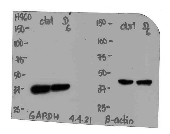

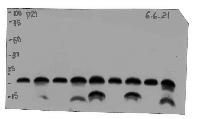

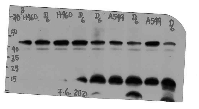


Fig. 2c

p21

p53

GAPDH

H460

A549

β-actin

H460

A549

Fig. 3b


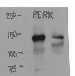

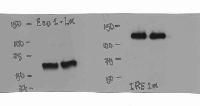

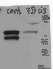

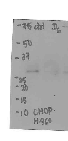

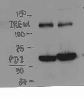

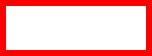


PERK

ERO1-L⍺

IRE-1⍺

GRP78

CHOP

PDI


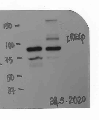


P-IRE-1⍺

Calnexin

Fig. 3d


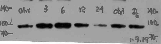


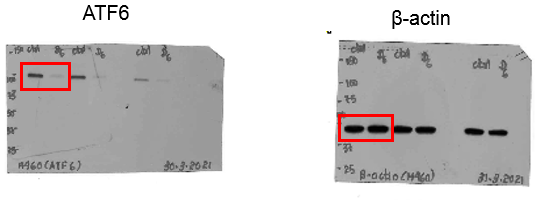


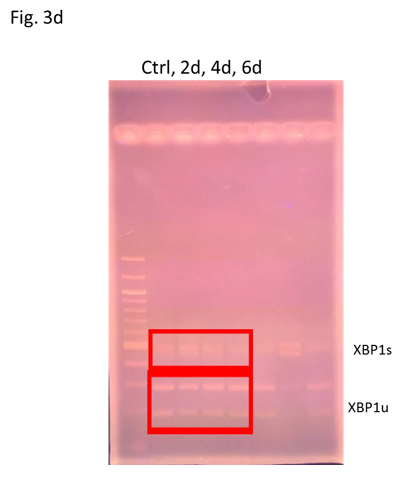


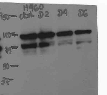

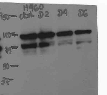

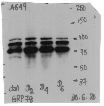

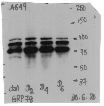

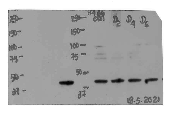

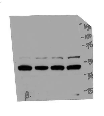


Fig. 4a

GRP78_H460

Calnexin_H460

GRP78_A549

Calnexin_A549

β-actin_H460

β-actin _A549


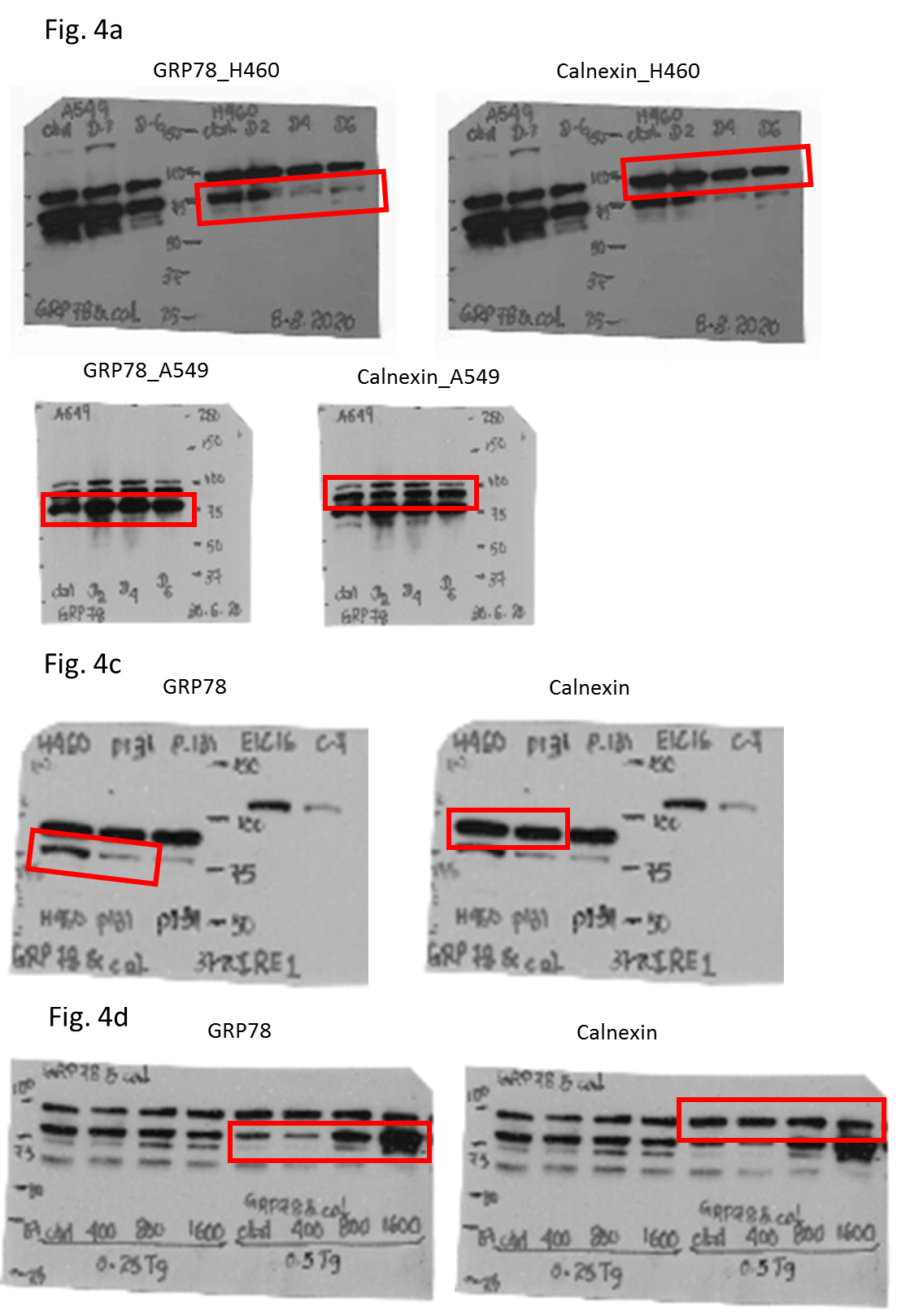


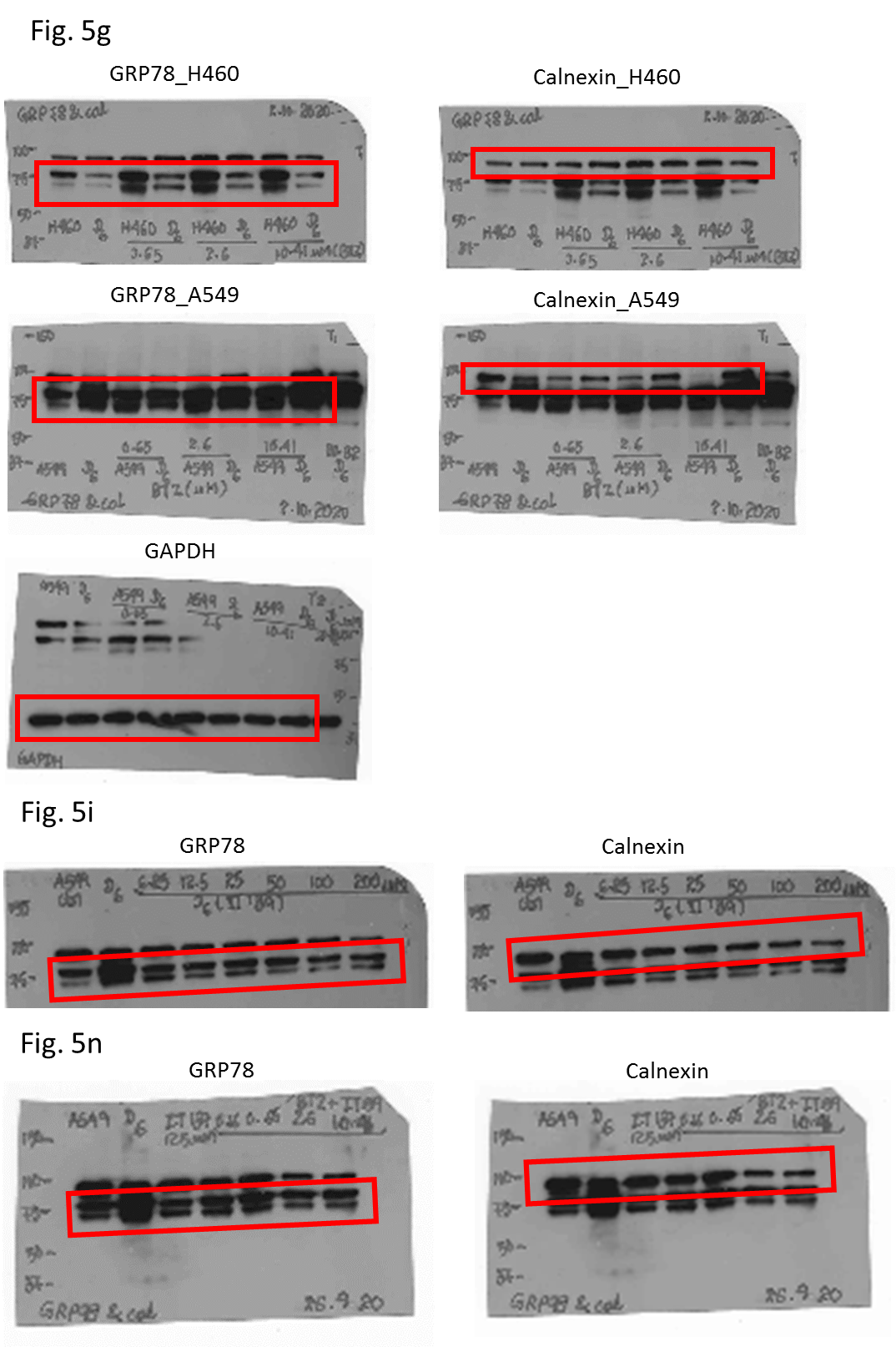

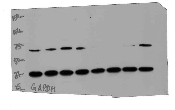


GAPDH_H460

GAPDH_A549


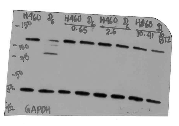


GAPDH


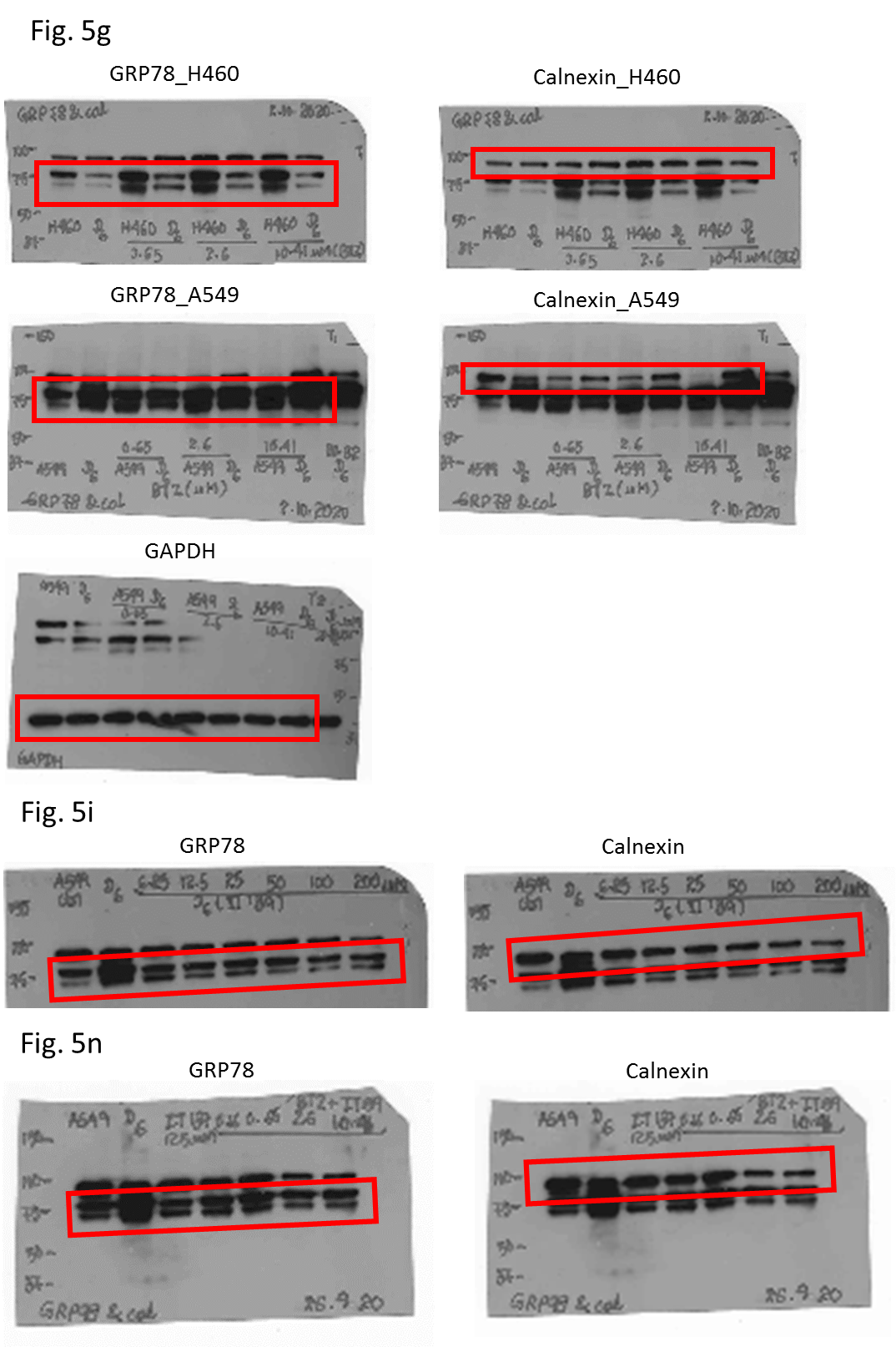


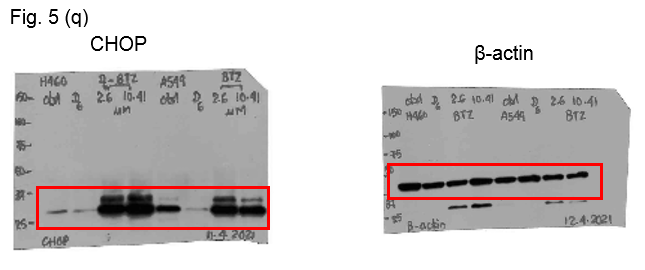


Fig. S1


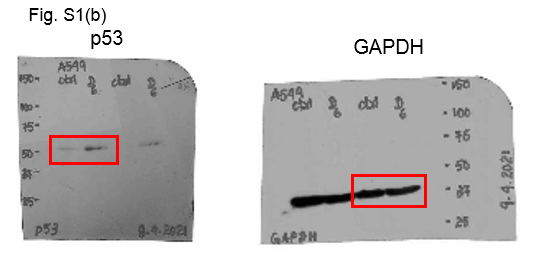


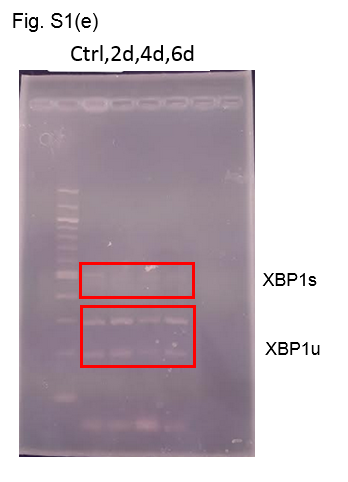


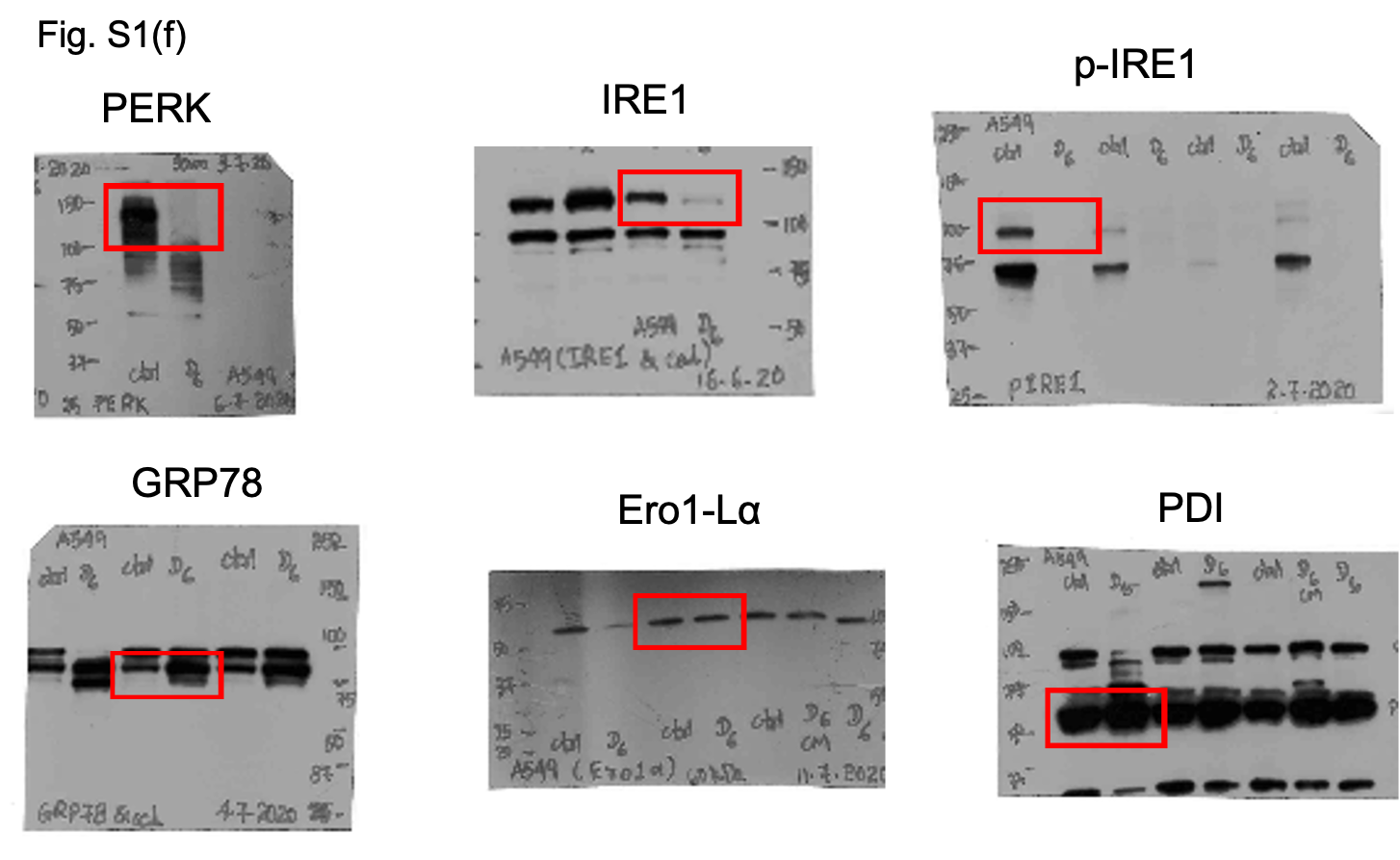


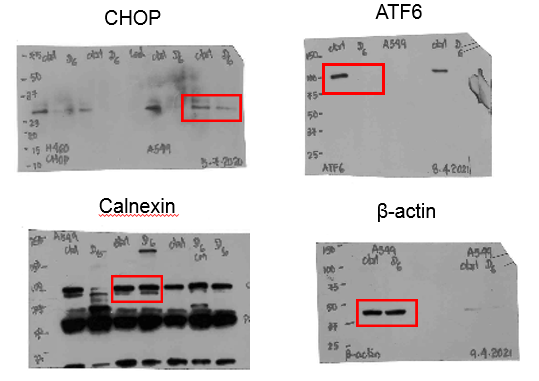


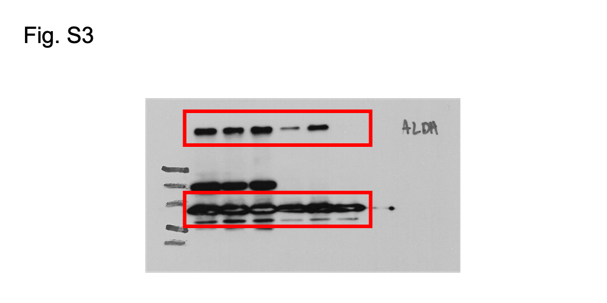


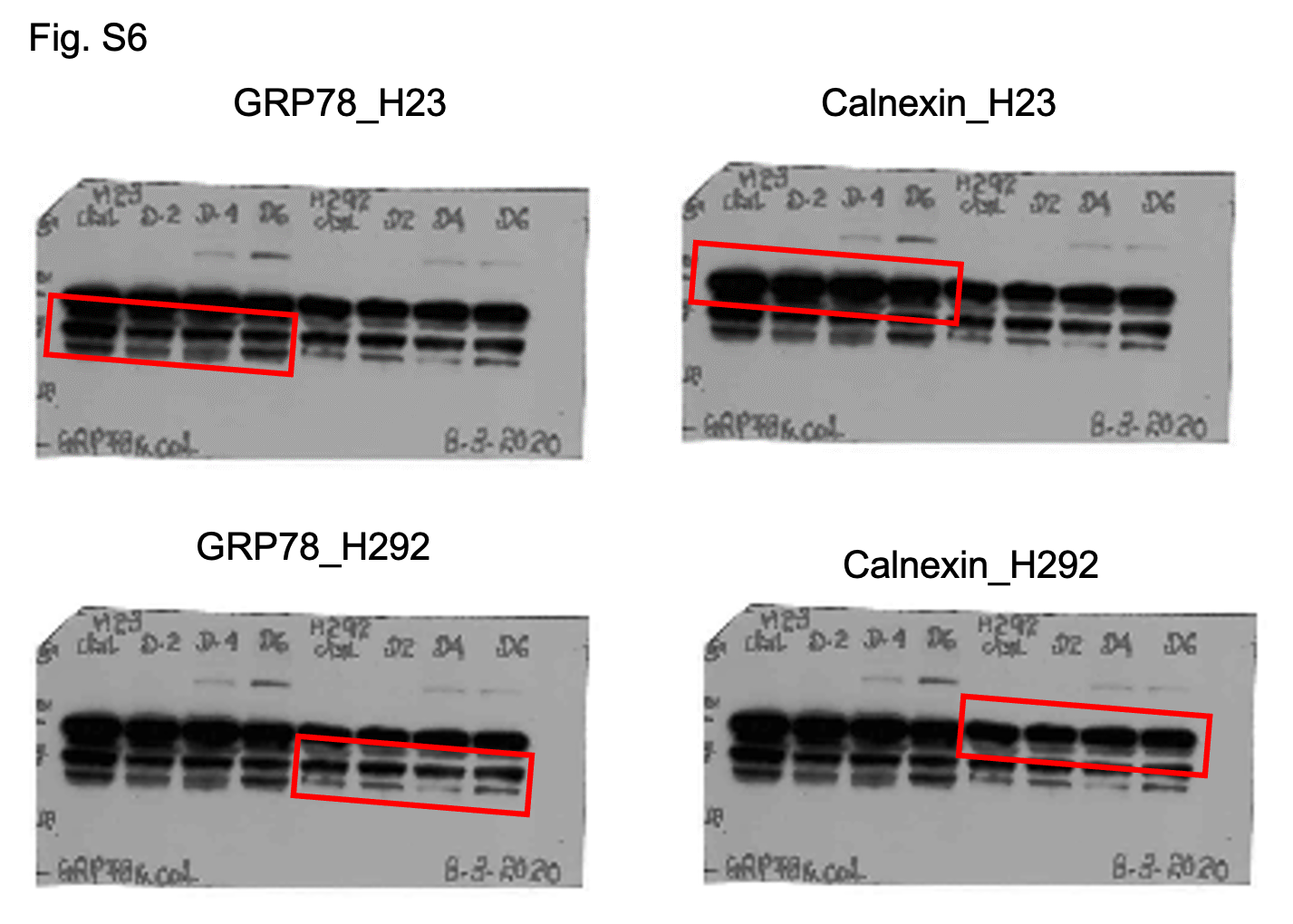


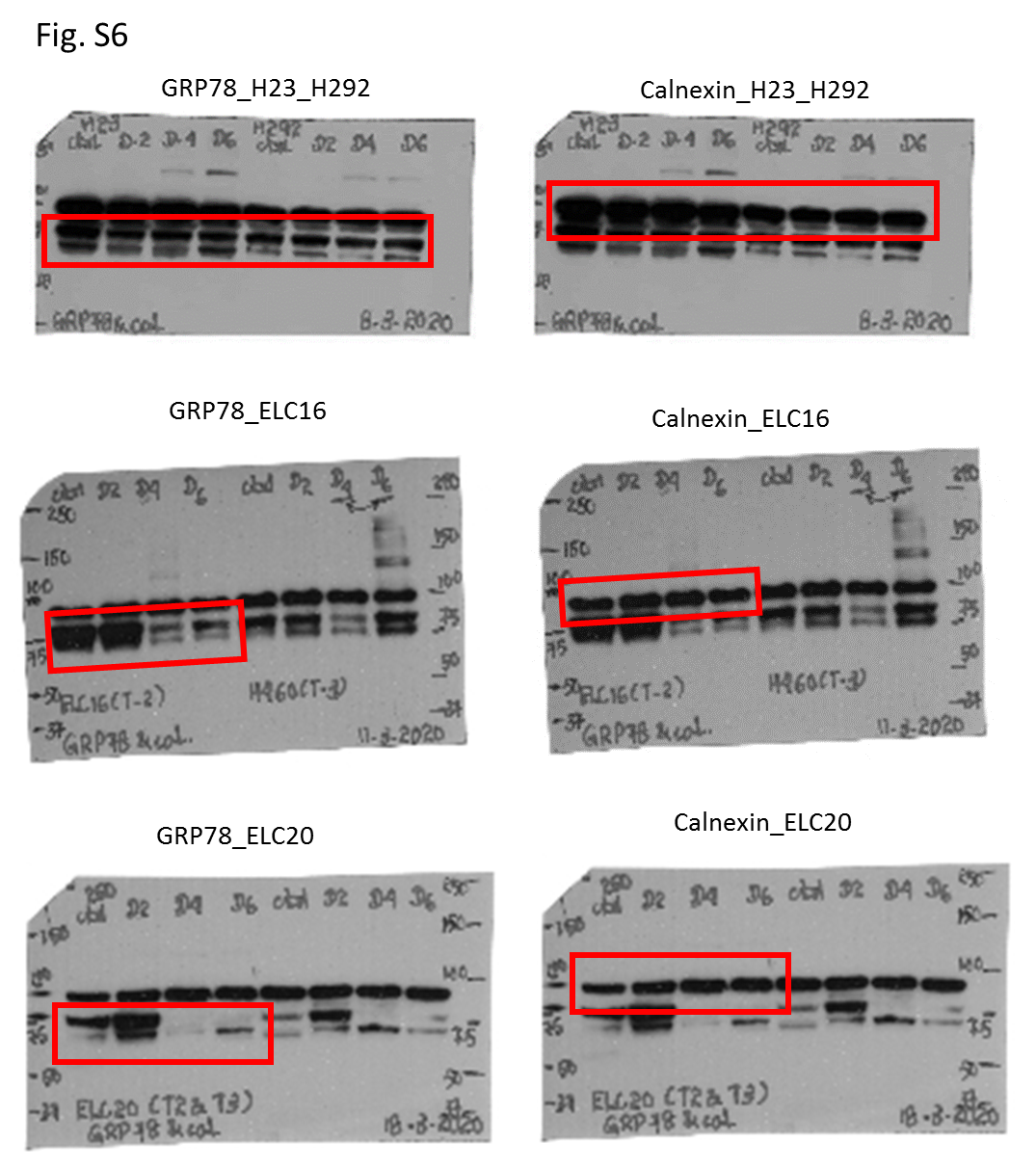


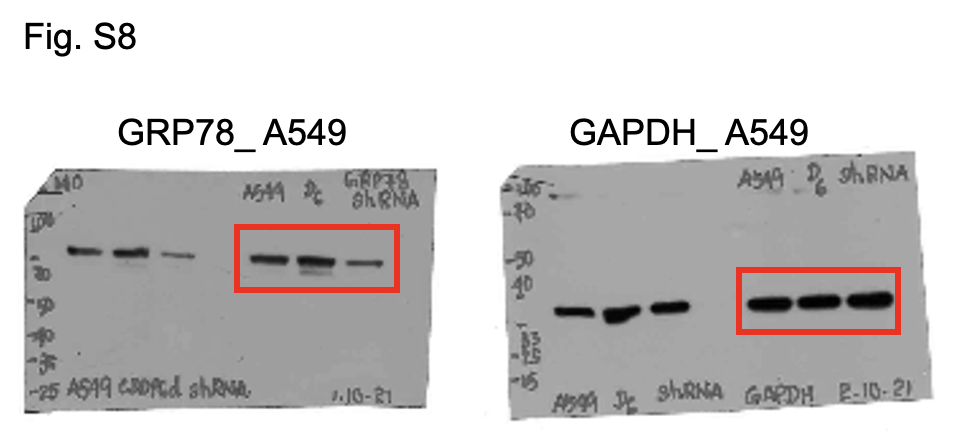

Supplement: Supplementary file 1 — Supplementary Information. [file 41598_2021_1540_MOESM1_ESM.docx]
